# Epstein-Barr virus lytic infection promotes activation of Toll-like receptor 8 innate immune response in systemic sclerosis monocytes

**DOI:** 10.1186/s13075-017-1237-9

**Published:** 2017-02-28

**Authors:** Antonella Farina, Giovanna Peruzzi, Valentina Lacconi, Stefania Lenna, Silvia Quarta, Edoardo Rosato, Anna Rita Vestri, Michael York, David H. Dreyfus, Alberto Faggioni, Stefania Morrone, Maria Trojanowska, G. Alessandra Farina

**Affiliations:** 1 Rheumatology, Boston University School of Medicine, Arthritis Center, 72 E. Concord Street, E-5, Boston, MA 02118 USA; 2 Department of Experimental Medicine, Sapienza University, Rome, Italy; 3 Istituto Italiano di Tecnologia, CLNS@Sapienza, Rome, Italy; 4 Department of Clinical Medicine, Sapienza University, Rome, Italy; 5 Department of Public Health, Sapienza University, Rome, Italy; 6 Department of Pediatrics, Yale, New Haven, CT USA

**Keywords:** Systemic sclerosis, EBV reactivation, Monocytes, Toll-like receptor 8, Innate immune response, IFN inducible genes

## Abstract

**Background:**

Monocytes/macrophages are activated in several autoimmune diseases, including systemic sclerosis (scleroderma; SSc), with increased expression of interferon (IFN)-regulatory genes and inflammatory cytokines, suggesting dysregulation of the innate immune response in autoimmunity. In this study, we investigated whether the lytic form of Epstein-Barr virus (EBV) infection (infectious EBV) is present in scleroderma monocytes and contributes to their activation in SSc.

**Methods:**

Monocytes were isolated from peripheral blood mononuclear cells (PBMCs) depleted of the CD19+ cell fraction, using CD14/CD16 negative-depletion. Circulating monocytes from SSc and healthy donors (HDs) were infected with EBV. Gene expression of innate immune mediators were evaluated in EBV-infected monocytes from SSc and HDs. Involvement of Toll-like receptor (TLR)8 in viral-mediated TLR8 response was investigated by comparing the TLR8 expression induced by infectious EBV to the expression stimulated by CL075/TLR8/agonist-ligand in the presence of TLR8 inhibitor in THP-1 cells.

**Results:**

Infectious EBV strongly induced TLR8 expression in infected SSc and HD monocytes in vitro. Markers of activated monocytes, such as IFN-regulated genes and chemokines, were upregulated in SSc- and HD-EBV-infected monocytes. Inhibiting TLR8 expression reduced virally induced TLR8 in THP-1 infected cells, demonstrating that innate immune activation by infectious EBV is partially dependent on TLR8. Viral mRNA and proteins were detected in freshly isolated SSc monocytes. Microarray analysis substantiated the evidence of an increased IFN signature and altered level of TLR8 expression in SSc monocytes carrying infectious EBV compared to HD monocytes.

**Conclusion:**

This study provides the first evidence of infectious EBV in monocytes from patients with SSc and links EBV to the activation of TLR8 and IFN innate immune response in freshly isolated SSc monocytes. This study provides the first evidence of EBV replication activating the TLR8 molecular pathway in primary monocytes. Immunogenicity of infectious EBV suggests a novel mechanism mediating monocyte inflammation in SSc, by which EBV triggers the innate immune response in infected cells.

**Electronic supplementary material:**

The online version of this article (doi:10.1186/s13075-017-1237-9) contains supplementary material, which is available to authorized users.

## Background

Systemic sclerosis (scleroderma; SSc) is a complex autoimmune disease characterized by immune abnormalities, vascular damage, and fibrosis predominantly involving the skin and lungs [1]. An aberrant innate immune response is suspected to activate both immune and non-immune effector cells in SSc, as evidenced by the presence of an interferon (IFN) signature in the affected tissues and the genetic predisposition toward genes linked to the IFN pathways [2, 3].

Monocytes/macrophages are known to play a crucial role in the innate immune process [4]. These cells have been found in SSc tissues, suggesting that monocytes/macrophages might be involved in the pathogenesis of the disease [5, 6]. Abnormalities in monocytes have been documented in SSc [7]. Studies have shown increased expression of several IFN-regulatory genes, including sialic acid-binding Ig-like lectin 1 Siglec1/CD169 (Siglec1), a marker of activated monocytes/macrophages, and numerous monocyte inflammatory cytokines in peripheral blood mononuclear cells (PBMCs) and sera from SSc patients, implicating dysregulation of the innate immune response in the activation of these cells [8–11]. However, what triggers and sustains monocyte activation in SSc remains unclear.

Although innate immunity is classically viewed as a first line of resistance against pathogens, little is known about pathogens as the source of the innate immune activation in SSc [12]. The expression of Epstein-Barr virus (EBV) lytic mRNA and proteins in PBMCs and skin of SSc patients has been associated with aberrant antibody response against EBV lytic antigens [13, 14], suggesting that EBV dysregulation may be more prevalent in SSc patients. The lytic form of EBV infection (infectious EBV) is detected by the host innate immune system. In this regard, monocytes have been shown to detect unmethylated viral genomes by Toll-like receptor (TLR)9 [15], suggesting that these cells participate in the innate immune control of EBV. Given these observations, we sought to investigate whether EBV infection in monocytes might contribute to their activation in SSc. Here, we demonstrate that EBV replicates in primary human monocytes several days post-infection (PI), and viral lytic genes strongly induce TLR8 expression and activation of the innate immune response in healthy donor (HD) and SSc monocytes infected with EBV. The presence of infectious EBV in SSc is substantiated by detecting the expression of EBV lytic mRNA and proteins in freshly isolated SSc monocytes, while it is absent in monocytes from HDs. Using microarray analysis, we show that SSc monocytes carrying infectious EBV exhibit a robust induction of the IFN signature and altered level of TLR8 expression compared to HDs. Our results suggest that monocyte activation in SSc may be a result of aberrantly controlled EBV infection.

## Methods

### Study subjects

All study subjects met the criteria for SSc as defined previously [16]. All subjects gave written informed consent. Subjects selected for this study, diffuse cutaneous SSc (dcSSc) patients (*n* = 53) and normal healthy donors (HD) (*n* = 34), are summarized in Table 1. All the patients and HDs included in the study were positive for EBV serology. All dcSSc patients included in the study were naive for immunosuppressive therapy (IT) or >6 months free of IT. Due to the variable number of PBMCs and monocytes obtained from the patients and controls, sets of experiments performed on the same subjects are indicated in Table 1.

**Table 1:** Clinical and demographic characteristics of the subjects enrolled in the study

	dcSSc*	HD
Number of subjects, *n*	53	34
Age in years, mean ± SE (range)	44 ± 1.8 (23–71)	41 ± 2.7 (23–65)
Female, %	96	94
mRSS**, mean ± SE	17 ± 0.7	–
Monocytes infected with recombinant EBV (qPCR, Western blot and immunofluorescence staining experiments), *n*	14	8
Monocytes freshly isolated (RT-PCR for DNA, qPCR, Western blot and immunostaining experiments), *n*	8	6
PBMCs (FACS analysis experiments), *n*	13	12
Skin biopsies (immunostaining experiments), *n*	18	8

*Inclusion criteria: disease duration <5 years; patients naïve for immunosuppressive therapy (IT) or >6 months free of IT

*dcSSc* diffuse cutaneous systemic sclerosis, *EBV* Epstein-Barr virus, *HD* healthy donor, *mRSS* modified Rodnan Skin Score, *PBMC* peripheral blood mononuclear cell, *qPCR* quantitative polymerase chain reaction, *RT-PCR* real-time polymerase chain reaction, *SE* standard error

### PBMC and monocyte isolation

Blood was collected from EBV-seropositive HD and dcSSc patients in CPT tubes designed for one-step cell separation (Becton Dickinson), and PBMCs were isolated as previously described [9]. After positive selection of CD19 cells (CD19+) using magnetic bead isolation (CD19+ selection EasySep, StemCell), monocytes were negatively selected using the Human Monocyte Enrichment Kit without CD16 Depletion (EasySep, StemCell). Purity of the monocyte population was determined by detection of CD163, CD16, and CD19 mRNA expression and using flow cytometry for the surface markers CD14 and CD163 (BD Pharmingen) (Additional file 1: Figure S1A and B).

### Virus preparation and EBV infection of monocytes and THP-1 cells

Viral stocks were obtained from culture supernatants of recombinant EBV-wt B95.8 genomes stably transfected into 293 cells (293-p2089) as previously described [13]. Before infection, monocytes from SSc patients and HDs were prepared by UV irradiation at 230 mW/cm^2^, using a Stratalinker XL1500 (Stratagene, Agilent technologies, Santa Clara, CA, USA). Given that human promyelomonocytic THP-1 cells (ATCC TIB-202) are an EBV-negative cell line, UV treatment was not performed on the cells primed for EBV infection. Monocytes and THP-1 cells were seeded at a density of 5 × 10^4^ cells/well in complete RPMI 1640 medium supplemented with 10% fetal bovine serum (FBS), and infected or mock infected with p2089-wtEBV as previously described [13].

### Cell treatment and reagents

Cells were seeded as indicated above; TLR-agonist stimulation was performed in complete medium with the following ligands (1 μg/ml): R837/imiquimod and CL264/9-benzyl-8-hydroxyadenine (for TLR7), CL075-thiazoloquinoline (for TLR8) (all from Invitrogen, Grand Island, NY, USA), IFNβ (500 U/ml; PBLinterferone), IFNγ (500 U/ml), and tumor necrosis factor (TNF)α (10 ng/ml) (all from R&D Systems). After 24 h of incubation, cells were harvested and stored in RNA lysis buffer for subsequent RNA isolation. When indicated, cells were treated for 24 h with CL075 or infected with EBV in the presence or absence of Bafilomycin-A1 (20 nM) (Sigma-Aldrich, St. Louis, MO, USA). At the indicated times PI or after ligand stimulation, proteins were harvested and analyzed by Western blot analysis.

### Nucleic acid extraction, RNA preparation and real-time polymerase chain reaction

DNA was extracted from monocytes using the Qiagen Extraction Kit (Qiagen, Valencia, CA, USA) and processed as previously described [13].

Total RNA from monocytes and B lymphocytes was extracted using an miRNAsy kit according to the manufacturer’s protocol (Quiagen) and processed as previously described [13]. The synthesized cDNAs were used as templates for quantitative real-time polymerase chain reaction (PCR) and primers used as described before [2, 13]. All real-time PCR was carried out using StepOnePlus Sequence Detector (Applied Biosystems, Life Technologies, Grand Island, NY, USA). The change in the relative expression of each gene was calculated using the ΔΔCt formula choosing a healthy human subject [2]. Target and control reactions were run on separate wells of the same quantitative PCR plate [2].

### Quantitative real-time PCR primers

Primers used to detect EBV genes and innate immune mediator genes were designed using Primer Express software (Applied Biosystems) and synthesized by Integrated DNA Technologies. The primers used for quanititative PCR, including 18S endogenous control, are summarized in Additional file 2 (Table S1). Expression of mRNA for the indicated genes was detected using SYBRGreen chemistry amplification (Applied Biosystems) as previously described [13]. To ensure specificity of the primer set, amplicons generated from the PCR reaction were analyzed for specific melting temperatures by using the melting curve software. For MCP1/CCL2, Siglec1, CXCL10, IRF5, IRF7, OAS3, TLR2, TLR3, TLR4, TLR7, TLR9, Myd88, IL-6, CD19, TNFα, LY6E, and 18S TaqMan primers and probe were used (Applied Biosystems).

### Microarray analysis

Microarray analysis of RNA (200 ng) was performed using standard protocols on Affymetrix Human Gene 2.0 ST arrays at the Boston University Microarray Core. All procedures were performed as described in the Affymetrix GeneChip user manual (www.affymetrix.com). CEL files were normalized to produce gene-level expression values using the implementation of the Robust Multiarray Average (RMA) in the affy package (version 1.36.1). Array quality was assessed by computing relative log expression (RLE) and normalized unscaled standard error (NUSE) using the *affyPLM* package (version 1.34.0). Differential expression was assessed using the moderated (empirical Bayesian) *t* test implemented in the *limma* package (version 3.14.4). Correction for multiple hypothesis testing was accomplished using the Benjamini-Hochberg false discovery rate (FDR). All microarray analyses were performed using the R environment for statistical computing (version 2.15.1).

### Western blot analysis

Monocytes were collected and washed with phosphate-buffered saline (PBS). Cell pellets were suspended in 2× SDS-Page buffer. Samples (30 μg) were heat denatured with reducing agent. Cellular extracts and blotted proteins were prepared and probed as previously described [13]. Blotted proteins were probed with each primary monoclonal antibody (mAb) respectively for BFRF1 [17, 18], BFLF2 [19], Zta/Zebra (Argene, bioMérieux, Inc. Durham, NC, USA), phospho-IRF7 (Cell Signaling Technology, Danvers, MA, USA), IRF7 (Abcam, Cambridge, MA, USA), TLR8 (Cell Signaling), and anti-β-actin-antibody (Sigma), and then probed with secondary antibody and visualized using a super signal chemiluminescence kit (Thermo Scientific, Pittsburg, PA, USA).

### Cytofluorimetric analysis

PBMCs were labeled with conjugated mouse mAb against human CD14 (PE-Cy7), CD16 (APC), CD163 (PE), CD169/siglec1 (FITC), and CD19 (APC/FITC) (BD Pharmingen). After incubation with antibodies, the cells were washed and then fixed with 2% formaldehyde. Cytofluorimetric analysis was performed using a BD FACSCanto II flow cytometer (Becton Dickinson, Mountain View, CA, USA). A total of 50,000 events were acquired for each sample. Data were processed using FlowJo software (Treestar, Inc., USA).

### Immunocytochemistry/immunohistochemistry

Monocytes were stained with mouse monoclonal antibodies against CD14-PE (BD Pharmingen), BFRF1 [18], gp-350-220 [13], rabbit TLR8 (Cell Signaling), and secondary-antibodies-Cy3-conjugated (Jackson IR, West Grove, PA, USA), Alexafluor-350 goat-anti mouse antibody (Invitrogen, Grand Island, NY, USA), or 488-labeling (Zenon kit; Invitrogen). Coverslips were mounted using Vectashield with DAPI (Vector Laboratories, Burlingame, CA, USA) and immunofluorescence staining was examined using a FluoView FV10i confocal microscope system (Olympus, Center Valley, PA, USA) at 488 nm (green), 594 nm (red), and 405 nm (blue). Paraffin sections of skin tissues were stained using mouse mAb against CD163 (AbD Serotec, Raleigh, NC, USA) and Zta/Zebra (Argene, bioMérieux, Inc., Durham, NC, USA) as previously described [13]. Immunohistochemical staining was examined using the Olympus BH2 microscope.

### Statistical analysis

All data are expressed as the mean ± SEM. Statistical comparisons between groups were tested by two-tailed *t* test. Significance was taken at *P* ≤ 0.05.

## Results

### De novo EBV replication occurs in primary cultured monocytes infected with EBV-p2089

Monocytes have been identified as a potential target for EBV infection [15]. The EBV genome has been detected in monocyte/macrophages from patients with several diseases, including rheumatoid arthritis and coronary diseases, supporting the ability of EBV to infect these cells in vivo [20–22]. Based on these observations, we first sought to investigate whether SSc monocytes were also capable of sustaining EBV replication.

**Fig. 1:**
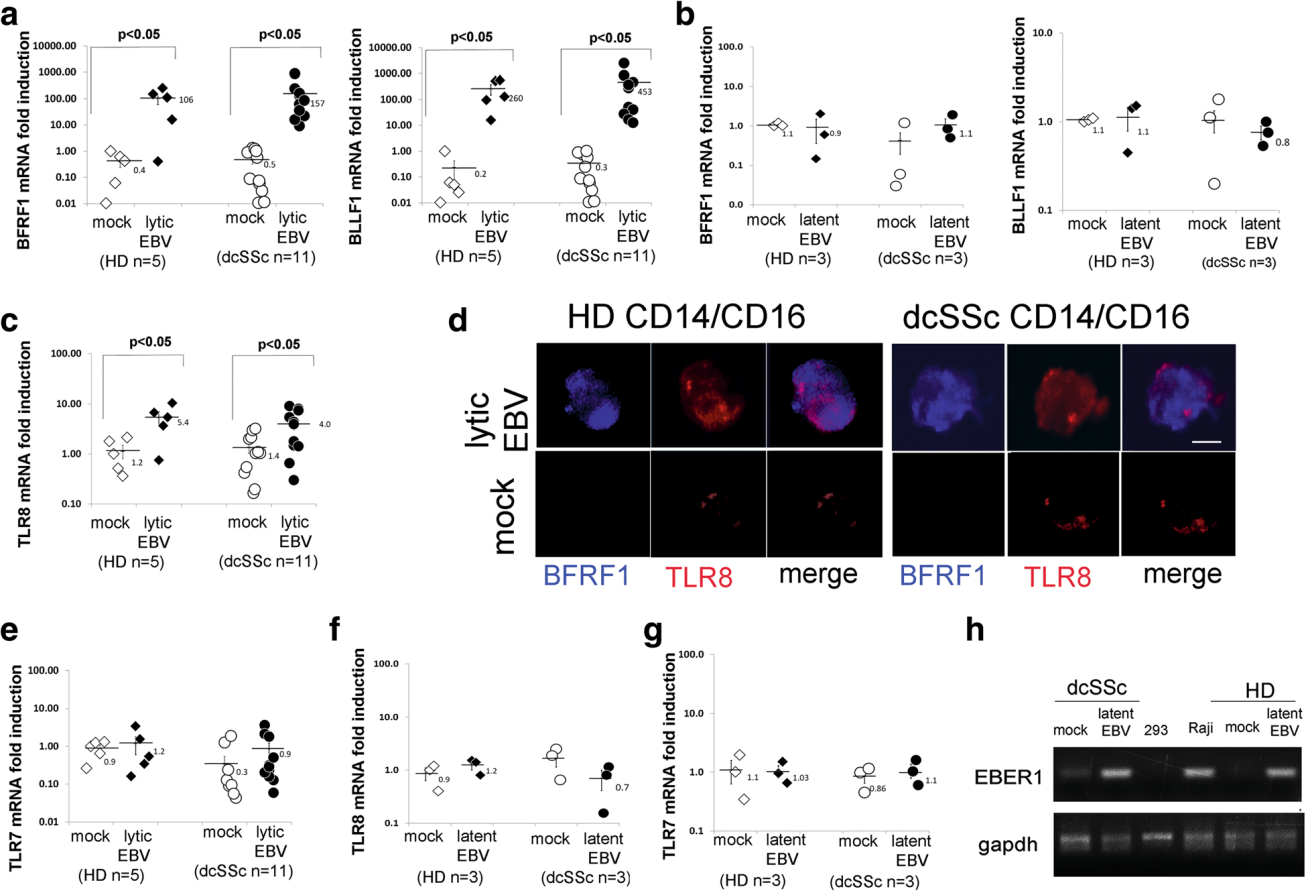
EBV replication modulates TLR8 expression in monocytes in vitro. Negative selected monocytes from dcSSc patients and HDs were infected with EBV. For total RNA and immunostaining, monocytes were harvested 5 days PI. **a**,**b**,**c**,**e**,**f,g** mRNA expression of EBV-lytic and TLR genes was analyzed by quantitative PCR. Results are expressed as fold-change induction normalized by one of the mock infected healthy controls. 18S rRNA was used as internal control. Bars represent the mean ± SEM. The two-tailed *t* test was used for statistical analysis. **d** Immunofluorescence double staining shows EBV-BFRF1-antigen (*blue*) and TLR8 (*red*) co-expression in monocytes from one representative dcSSc and HD infected with EBV-p2089. Original magnification 60×, *scale bar* = 5 μm. **h** PCR products of EBV DNA in monocytes from representative dcSSc and HD; DNA from Raji-EBV-positive cells and DNA from 293 were used as positive control and negative control, respectively. GAPDH used as internal control. **b**, **e**, **f** and **h** refer to monocytes expressing EBV-p2089 latent infection. *dcSSc* diffuse cutaneous systemic sclerosis, *EBV* Epstein-Barr virus, *HD* healthy donor, *TLR* Toll-like receptor

Circulating monocytes were isolated from HDs and SSc patients with diffuse cutaneous disease (dcSSc). UV-treated monocytes from 14 SSc patients and 8 HDs were infected with EBV recombinant virus (EBV-p2089). Active EBV infection was detected in monocytes from 11 SSc patients and 5 HD, while it did not occur in 3 SSc patients and 3 HDs 5 days PI (Fig. 1a and b). Specifically, we found that EBV/early-lytic (BFRF1) and EBV/late-lytic (BLLF1) genes were significantly upregulated in EBV-p2089-infected monocytes in the majority of dcSSc (*n* = 11) and HDs (*n* = 5), 5 days PI (Fig. 1a). Since the EBV/early-lytic BFRF1 protein is implicated in nucleo-capsid egress and EBV/late-lytic BLLF1/gp350-220 is expressed on the virion envelope and on EBV producer cells, these results indicate that de novo active viral infection occurs in monocytes, and that most dcSSc and HD monocytes are able to sustain efficient EBV replication in vitro.

### EBV replication induces TLR8 and innate immune mediators in infected monocytes

Given that EBV replicates in primary cultured monocytes, we next evaluated whether infectious EBV might trigger the innate immune response in EBV-p2089-infected monocytes that were previously UV inactivated. De novo viral replication strongly induced expression of TLR8 mRNA in lytic-infected cells (Fig. 1c), and BFRF1/lytic protein was associated with monocytes expressing high levels of TLR8 (Fig. 1d), suggesting that induction of TLR8 occurs in EBV-infected monocytes. Given that TLR7 together with TLR8 represent the two nucleic acid receptors detecting ssRNA and that TLR3 recognizes dsRNA, we sought to evaluate whether TLR7 and/or TLR3 could also be induced by EBV in infected monocytes. Unexpectedly, EBV-lytic mRNAs did not increase TLR7 expression in the infected cells (Fig. 1e), while TLR3 was only induced in monocytes from two HDs and one dcSSc patient (Additional file 1: Figure S2A). Moreover, we also found that EBV-p2089 induced expression of TLR9 in monocytes from a few HDs and dcSSc patients (Additional file 1: Figure S2B), confirming the previous observation [23]. No significant induction of TLR2 was found in EBV-p2089-infected monocytes (Additional file 1: Figure S2C).

EBV exploits two modes of infection: latent state and lytic replication [24]. Further supporting the linkage between viral replication and activation of the innate immune response, TLR8 or TLR7 expression was not induced in monocytes latently infected by EBV, as confirmed by the lack of infectious EBV in the cells harboring EBV-p2089 DNA (Fig. 1f and g, and h showing one representative dcSSc patient and HD). Notably, the naive EBV genome was expressed in the majority of dcSSc (7/8) and in some (3/6) HD monocytes (Table 2), suggesting that the prevalence of EBV DNA is higher in monocytes from dcSSc patients compared to HDs. Together, these findings suggest that newly lytic EBV mRNA is detected by TLR8 in infected HD and dcSSc monocytes.

**Table 2:** EBV gene expression and DNA in circulating monocytes and in skin macrophages of dcSSc patients

EBV protein/DNA	Number of dcSSc patients positive/tested	Number of HDs positive/tested
BFRF1* (monocytes)	3/8	0/6
BFLF2* (monocytes)	4/8	0/6
EBER1 DNA** (monocytes)	8/8	3/6
EBER1 DNA** (B-lymphocytes)	8/8	8/8
BZLF1/Zebra monocytes mRNA ***	4/8	0/6
CD163+/macrophages***	6/18	0/8

*Tested by Western blot

**Tested by real-time polymerase chain reaction

***Tested by immunohistochemistry on skin sections

*dcSSc* diffuse cutaneous systemic sclerosis, *EBV* Epstein-Barr virus, *HD* healthy donor

**Fig. 2:**
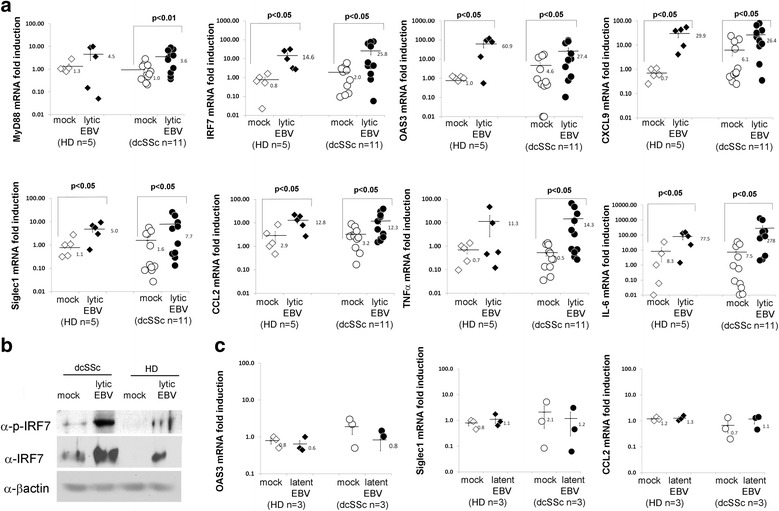
EBV lytic genes modulate the expression of innate immune mediators, IFN-inducible genes, and proinflammatory cytokines in EBV-p2089-infected monocytes. **a,c** mRNA expression of indicated innate immune mediators and cytokines was evaluated by quantitative PCR in negative selected CD14/CD16 monocytes infected with EBV-p2089, 5 days PI. Results are expressed as the fold-change induction normalized by one of the mock infected healthy controls. 18S ribosomal RNA levels used as internal control. Bars represent the mean ± SEM. **b** Western blot analysis was performed to determine IRF7 protein (phosphorylated and total) levels in cell lysates of mock- and EBV-infected monocytes from one representative dcSSc patient and HD, 5 days PI. β-actin was used as loading control. Fold-changes shown on the graph are normalized to mRNA expression by one of the mock infected healthy controls. Bars represent mean ± S.E.M. The two-tailed *t* test was used for statistical analysis. *dcSSc* diffuse cutaneous systemic sclerosis, *EBV* Epstein-Barr virus, *HD* healthy donor, *IL* interleukin, *Siglec1* sialic acid-binding Ig-like lectin 1 Siglec1/CD169, *TNF* tumor necrosis factor

To further characterize the TLR8 innate immune response induced by viral/lytic genes, innate immune mediators were examined in EBV-p2089-infected monocytes. MyD88 and IRF7 mRNA, as well as IRF7 protein (phosphorylated and total), expression was induced in the majority of infected monocytes from dcSSc patients and HDs (Fig. 2a and b). Since IRF7 is required to induce the innate antiviral response characterized by IFNs, IFN-regulated genes, and inflammatory cytokines [25], we next determined whether lytic EBV might induce in EBV-p2089 infected monocytes the same inflammatory genes found to be upregulated in dcSSc monocytes. We found that expression of CXCL9, OAS3, Siglec1, and cytokines, including CCL2, TNFα, and interleukin (IL)-6, were robustly increased in EBV-p2089-infected monocytes (Fig. 2a). In contrast, no upregulation of innate immune mediators and proinflammatory cytokines was observed in latently infected monocytes (Fig. 2c and Additional file 1: Figure S3). To better appreciate the magnitude of innate immune mediators induced by infectious EBV in infected cells, in a parallel experiment, monocyte fractions not exposed to the virus were stimulated with CL075, a well-established TLR8/ligand positive control [26, 27]. We found that TLR8 ligand stimulation significantly induced the expression of Siglec1 and CCL2 genes in dcSSc and HD monocytes, comparable to the induction observed by infectious EBV in EBV-infected cells (Additional file 1: Figure S4). Statistically, no significant differences were observed in Siglec1 and CCL2 gene induction in TLR8-stimulated and EBV-infected monocytes.

Taken together, these results suggest that TLR8 expression and the innate immune inflammatory response are induced by de novo infectious EBV in EBV-infected dcSSc and control monocytes.

### Infectious EBV induces TLR8 protein expression in a TLR8-dependent and TLR8-independent manner in infected THP-1 cells

**Fig. 3:**
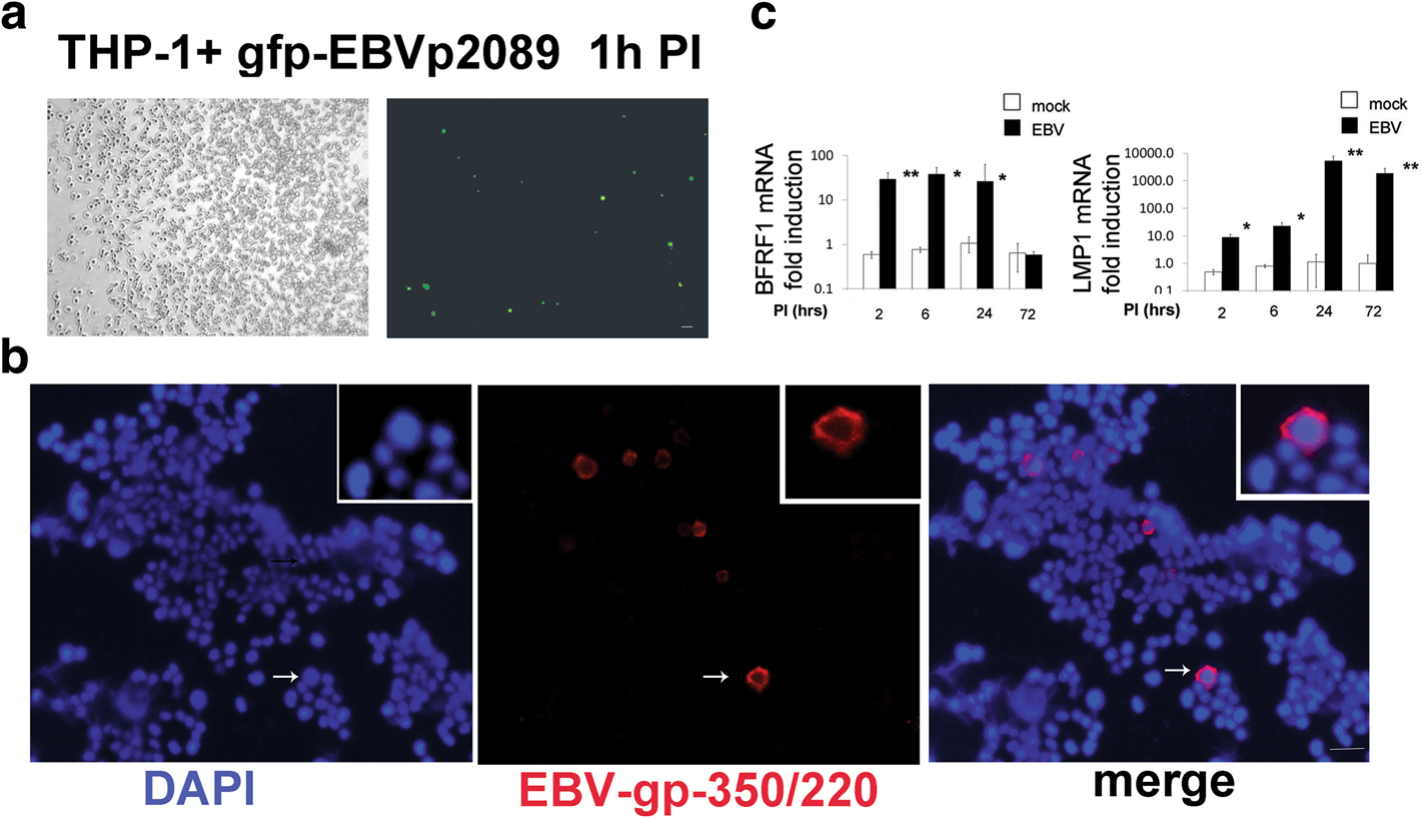
EBV lytic genes are expressed in THP-1 cells. THP-1 cells were infected with EBV. Total RNA and proteins were harvested at indicated time points, processed, and analyzed by quantitative PCR and immunofluorescence. **a** Detection of EBV-p2089-GFP in THP-1 after 1 h PI (*left*: phase-contrast light microcopy, *scale bar* = 0.1 mm). **b** Double-indirect immunofluorescence staining of THP-1 cells co-stained with anti-EBV/gp-350 antibodies. Diaminidino-2-phenylindole (*DAPI*) was used as counterstaining for the nuclei. Insert: Higher magnification view for detail (*arrow*) Original magnification 10×; *scale bar* = 10 μm. **c** mRNA expression of indicated genes and proteins in mock-infected and EBV-infected THP-1 cells at indicated time points. Data are expressed as the fold-change normalized to mRNA expression in a mock-infected sample for each time point. Bars represent mean ± SEM from three separate experiments. *P* values calculated using two-tailed *t* test; *P < 0.05, **P < 0.01. *EBV* Epstein-Barr virus, *PI* post-infection

**Fig. 4:**
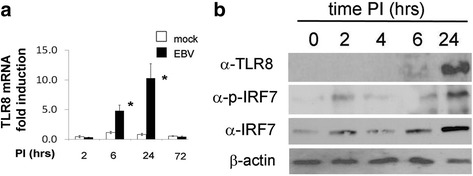
TLR8 modulation during EBV infection in THP-1 cells at different time points. THP-1 cells were infected with EBV. Total RNA and proteins were harvested at indicated time points, processed, and analyzed by quantitative PCR and Western blot. **a** TLR8 mRNA expression in mock-infected and EBV-infected THP-1 cells at indicated time points. Data are expressed as the fold-change normalized to mRNA expression in a mock-infected sample for each time point. Bars represent mean ± SEM from three separate experiments. *P* values calculated using two-tailed *t* test; *P < 0.05. **b** Western blot analysis was performed to determine TLR8 and IRF7 protein (phosphorylated and total) levels in cell lysates at indicated time points post-infection (*PI*). β-actin was used as loading control. *EBV* Epstein-Barr virus, *TLR* Toll-like receptor

To elucidate the role of EBV in viral-mediated TLR8 signaling in monocytes, THP-1 monocytic cell line was infected with EBV-p2089. UV treatment did not induce TLR8 mRNA expression in THP-1 UV treated cells (Additional file 1: Figure S5). Given that THP-1 cells are negative for EBV genome expression, UV treatment was not performed on the cells primed for EBV infection. After the exposure to recombinant EBV, 10–20% cell/field of THP-1 cells showed cellular p2089-GFP fluorescence at 1 h PI (Fig. 3a). EBV gp-350/late-lytic proteins were expressed in 2–3% of the cells 1 h PI (Fig. 3b). Kinetics of EBV infection showed a significant increase of the BFRF1/early-lytic gene over 2–24 h, while the LMP-1/latent gene was significantly induced up 72 h PI (Fig. 3c). Increased expression of TLR8 mRNA was induced in EBV-infected cells by 6 h with maximal induction at 24 h PI (Fig. 4a). In contrast, no induction of TLR8 mRNA was observed in latently infected cells at 72 h PI (Fig. 4a). TLR8 protein expression was also induced in THP-1-EBV-infected cells (Fig. 4b). In conjunction with TLR8, increased expression of IRF7 (phosphorylated and total) was also observed in THP-1-infected cells (Fig. 4b). Intriguingly, we found that IRF7 expression was also induced at 2 h PI, in agreement with the previous studies showing that EBV induces an early innate immune response in monocytes [21, 28].

**Fig. 5:**
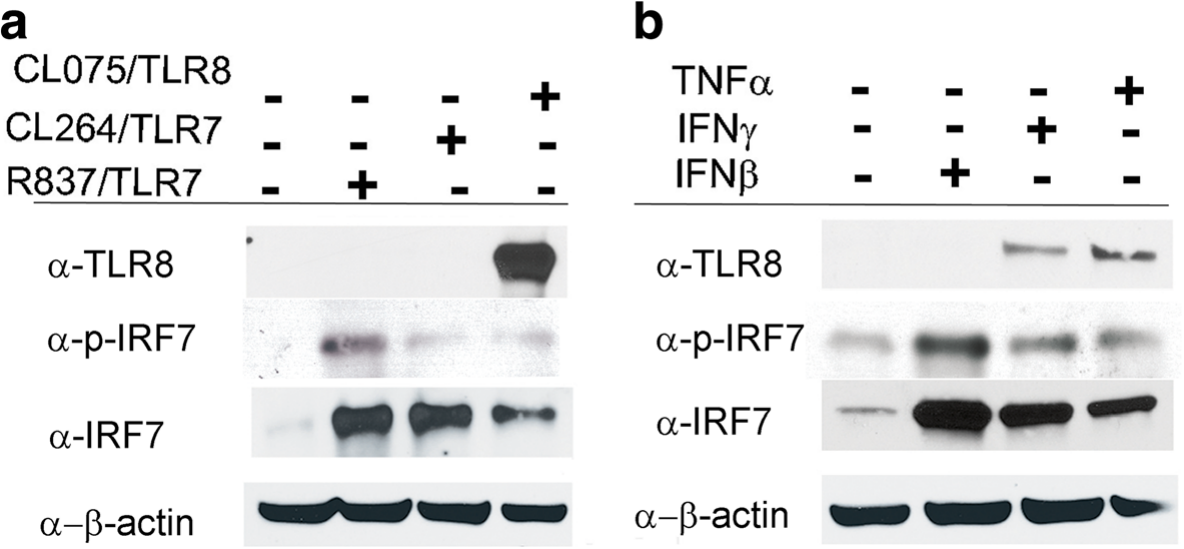
CL075/TLR8 synthetic agonist ligand, IFNγ, and TNFα induce TLR8 expression. Cellular lysates from THP-1 cells treated with TLR synthetic ligand: **a** CL264 (adenine analog), R837 (Imiquimod), CL075 (3 M002), or **b** IFNβ, IFNγ, and TNFα or untreated were extracted after 24 h and analyzed by Western blot. Representative immunoblots of TLR8 and IRF7 expression in whole cell lysates. β-actin was used as loading control. *IFN* interferon, *TNF* tumor necrosis factor; *TLR* Toll-like receptor

**Fig. 6:**
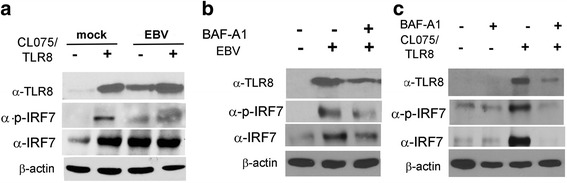
EBV-induced TLR8 is partially mediated by TLR8. THP-1 cells were infected with EBV in **a** presence/absence of CL075/TLR8 agonist ligand and **b,c** with/without bafilomycin-A1 (*BAF-A1*). Proteins were assayed 24 h after infection and CL075/TLR8 synthetic ligand stimulation as indicated. Representative immunoblots of TLR8 and IRF7 expression are shown. β-actin was used as loading control. *EBV* Epstein-Barr virus, *TLR* Toll-like receptor

Viral ssRNA has been identified as a natural agonist of TLR7 and TLR8 [27], and distinct RNA motifs selectively activate TLR7- and/or TLR8-mediated innate immune responses [26]. THP-1 cells were stimulated with TLR ligands R837 and CL264 (for TLR7) and CL075 (for TLR8). We found that activation of TLR8 by CL075 induced expression of TLR8 and to a lesser extent IRF7 proteins (Fig. 5a). In contrast, activation of TLR7/agonist ligands failed to induce expression of TLR8 protein (Fig. 5a). IRF7 protein was increased by stimulation of TLR7 ligands (Fig. 5a), IFNβ, IFNγ, and TNFα, and to a lesser extent by TLR8 ligand (Fig. 5b). Moreover, we observed that IFNγ and TNFα also induced TLR8 protein expression in THP-1 cells, although to a lesser extent than selective TLR8 ligand stimulation (Fig. 5b). Consistent with this finding, virally induced TLR8 stimulation was mostly comparable to the induction observed by TLR8/selective agonist/ligand in THP-1 cells (Fig. 6a, Additional file 1: Figure S6A). Notably, the combination of EBV and CL075-TLR8/ligand was more effective in inducing TLR8 (Fig. 6a).

We next investigated whether the TLR8 activation by EBV is mediated in a TLR8-dependent manner in infected THP-1 cells. We found that bafilomycin-A1, an inhibitor of endosomal acidification and autophagy [29], partially decreased TLR8 and IRF7 expression in THP-1 EBV-infected cells, although it strongly reduced TLR8 and IRF7 expression in CL075/TLR8 stimulated cells (Fig. 6b and c, Additional file 1: Figure S6B). Altogether, these results suggest a role on the TLR8 response in mediating EBV active infection.

### IFN-regulated genes and TLR8 are upregulated in freshly purified dcSSc carrying infectious EBV

**Fig. 7:**
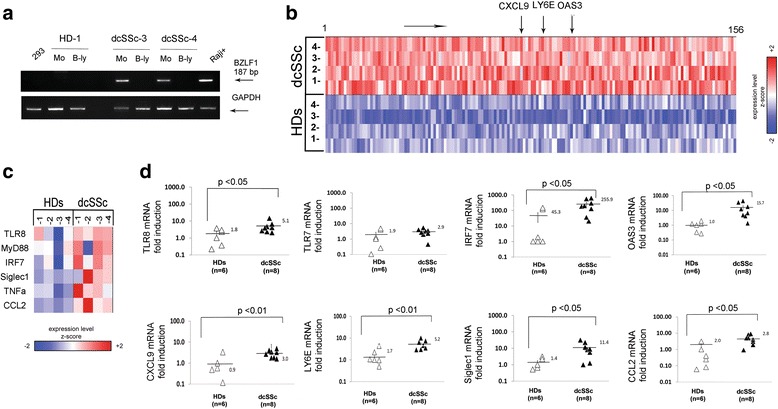
TLR8 and IFN-inducible gene signature in freshly isolated dcSSc monocytes carrying infectious EBV. **a** PCR products of EBV-lytic gene in freshly isolated monocytes (*Mo*) and B lymphocytes (*B-ly*) from two representative diffuse cutaneous systemic sclerosis (*dcSSc*) patients and one healthy donor (*HD*); 293 and Raji cells were used as negative and positive controls, respectively. **b** Heatmap showing the expression of the 156 most significantly upregulated genes (moderated *t* test) FDR *q* < 0.25 in freshly isolated lytic/EBV-positive dcSSc compared to HD monocytes. Colors are scaled within each gene so that *red* and *blue* indicate expression of values ≥2 standard deviations above and below, respectively, the mean (*white*) computed across all samples. **c** Heatmap of the expression of select genes, with colors scaled in the same manner as in panel **b**. **d** mRNA expression of indicated genes. Results are expressed as the fold-change normalized to mRNA expression in a single sample from HDs. Levels of 18S ribosomal rRNA were used as an internal control. Bars represent the mean ± SEM. Values were calculated using two-tailed *t* test

Consistent with our finding that the newly lytic EBV mRNA possibly activates the TLR8 innate immune response in infected monocytes, we next asked whether viral lytic mRNA would be detected in dcSSc monocytes. Due to the possibility of altering endogenous EBV, UV treatment was not performed on these sets of freshly isolated monocytes. We found expression of BZLF1/EBV-early-lytic-transactivator mRNA in freshly isolated monocytes from four dcSSc patients (*n* = 4/8) (Fig. 7a and Table 2). cDNA sequencing confirmed BZLF1 specificity of the real-time PCR products (data not shown). In contrast, the corresponding B-lymphocyte fraction from dcSSc patients and the monocyte and B-lymphocyte fractions from HDs did not express the BZLF1 gene (Fig. 7a), although EBV DNA was detected in all B lymphocytes and monocytes from dcSSc patients, as well as in B lymphocytes (*n* = 6/6) and in monocytes (*n* = 3/6) from HDs (Additional file 1: Figure S7, and Table 2). Together, these results suggest that while B lymphocytes carry EBV latent infection, freshly isolated dcSSc monocytes sustain the viral replication.

With the aim of determining whether the innate immune response seen upregulated in EBV-p2089-infected monocytes would also be increased in freshly isolated dcSSc EBV-infected monocytes, the gene expression profile was examined in dcSSc monocytes tested positive for infectious EBV compared to HDs. Remarkably, the microarray analysis revealed that most of the EBV-induced IFN-regulated genes were significantly upregulated (FDR *q* < 0.25) in lytic/EBV-positive dcSSc compared to HD monocytes (Fig. 7b, and Additional file 3: Table S2). To a lesser extent, expression of TLR8, MyD88, IRF7, Siglec1, CXCL10, CCL2, and TNFα was also increased in dcSSc compared to HD monocytes (Fig. 7c). mRNA Affymetrix data (GEO: GSE86984) are available at the public repository Gene Expression Omnibus. Microarray results were confirmed by quantitative PCR in a larger group of dcSSc patients (*n* = 8) and HDs (*n* = 6) (Fig. 7d), further supporting that TLR8 and IFN-regulated genes were significantly increased in dcSSc compared to HD monocytes. No difference in expression of TLR7, TLR3, and TLR9 was observed in dcSSc compared to HD monocytes (Fig. 7d and data not shown).

These results substantiate the observation that infectious EBV is associated with the activation of TLR8 and IFN proinflammatory response in dcSSc monocytes.

### Segregation of Siglec1 expressing subsets on CD14/CD16 dcSSc monocytes

Circulating monocytes defined by different expression levels of CD14 and CD16 surface markers are known to possess distinct phenotypes and functions [30]. Moreover, altered proportions and conversion of monocyte subsets CD14+/CD16– into CD16+ occur in most autoimmune diseases [31]. Given that EBV lytic infection may occur in a distinct subset of monocytes, which could become positive for the expression of the Siglec1, we next sought to determine whether Siglec1-positive cells might be ascribed to a specific population of ex vivo dcSSc monocytes.

**Fig. 8:**
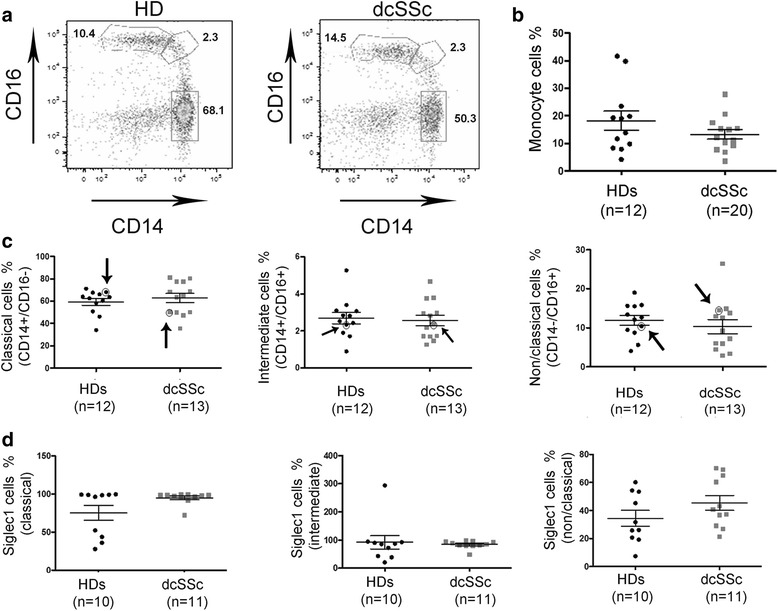
Monocyte subsets and Siglec1 expression in dcSSc patients and HD. **a** Representative monocyte subsets from PBMCs of diffuse cutaneous systemic sclerosis (*dcSSc*) patients and healthy donors (*HDs*) defined by the expression of CD14 and CD16 and gating by FACS analysis as CD14+/CD16– (classical, 68% HD), CD14+/CD16+ (intermediate, 2.3% HD), and CD14–/CD16+ (non-classical, 10.4 HD). **b** Number of monocytes (%) in HD compared to dcSSc patients. **c** Frequencies of classical, intermediate, and non-classic monocyte subsets in HD and dcSSc patients. **d** Expression of Siglec1 in the indicated subsets. *P* values calculated using two-tailed *t* test. *Arrows* and *circles* indicate the representative sample for each class of monocytes shown in panel **a**

Using FACS analysis, monocytes were identified as previously reported in three subsets: CD14+/CD16– (classical), CD14+/CD16+ (intermediate), and CD14–/CD16+ (non-classical) monocytes (Fig. 8a) [31, 32]. We did not find any significant difference between the percentage of total monocytes or subsets from dcSSc patients compared to HDs (Fig. 8b and c). Interestingly, we found a trend towards an increased number of non-classical monocytes expressing Siglec1 and a slight increase in classical monocytes, but no difference in the intermediate subset (Fig. 8d). These results could suggest that Siglec1-activated monocytes are generally increased in the CD14–/CD16+ non-classical subset and to a lesser extent in the classical subset in dcSSc patients compared to HDs.

### EBV/lytic proteins are present in circulating monocytes and in skin macrophages from patients with dcSSc

**Fig. 9:**
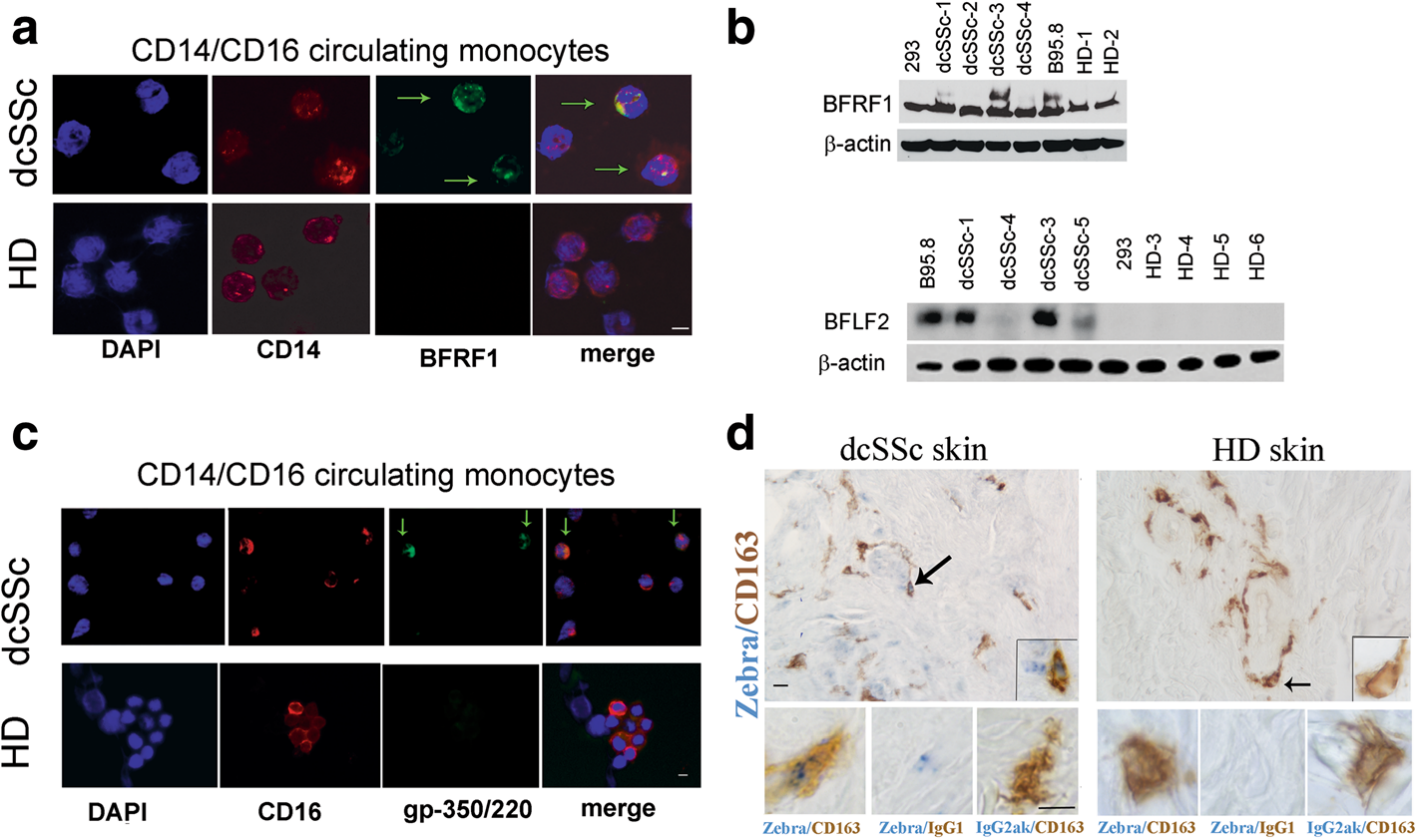
EBV-lytic proteins are expressed in dcSSc circulating monocytes and dcSSc skin macrophages. Immunofluorescence and Western blot analysis of CD14/CD16 monocytes freshly purified from PBMCs by negative selection, and immunohistochemistry of skin sections. **a**, **c** Double immunofluorescence shows the presence of EBV-BFRF1 antigen (*green*) in CD14+ (*red*), and EBV-gp-350-220 (*red*) in CD16+ (*green*) from two representative diffuse cutaneous systemic sclerosis (*dcSSc*) patient and two healthy donors (*HD*). *Green arrows* indicate monocytes expressing EBV/lytic proteins. Original magnification 60×. *Scale bar* = 10 μm. **b** Western blot analysis of EBV-BFRF1 and EBV-BFLF2 lytic proteins in cell lysates of dcSSc and HD monocytes; B95-8/EBV-expressing cells and 293 were used as positive and negative control, respectively. β-actin used as loading control. **d** Representative immunohistologic images of CD163+ macrophages (*brown*) and EBV/lytic protein Zebra + (*blue*) cells in skin sections from dcSSc and HD. Original magnification 2× (*upper panels*) and 10× (*inserts* and *lower panels*). The *arrows* indicate monocytes represented in the high magnification inserts. *Scale bars* = 5 μm

To identify whether infectious EBV might be ascribed to a specific population of ex vivo dcSSc monocytes, CD14 and CD16 surface markers were evaluated in freshly isolated EBV-infected cells [30]. EBV/lytic proteins were expressed in a subset of CD14+ and CD16+ monocytes from dcSSc (Fig. 9a and c, arrows; Fig. 9b shows four representative dcSSc patients) (Table 2). In contrast, we did not find any EBV expression in circulating monocytes from six HDs (Fig. 9b shows four representative HDs).

Given that dcSSc circulating monocytes are carrying infectious EBV, and increased macrophages have been shown in the perivascular areas of SSc skin [8, 33], we next sought to determine whether skin macrophages from dcSSc patients express EBV/lytic proteins. An estimated 2–3% cell/field double positive cells for EBV/nuclear/immediate-early/lytic protein BZLF1/Zebra (Zebra+) and monocyte/macrophage surface/antigen-CD163 (Zebra+/CD163+) were found in 6/18 dcSSc skin biopsies (Fig. 9d, Table 2). As expected, Zebra + staining was found in the nuclei of the infected cells, while CD163 staining was spread around the cell (Fig. 9d, lower panel, high magnification). We did not find any expression of Zebra + or double Zebra+/CD163+ macrophages in the skin from HDs (Fig. 9d). In general, this finding suggests that skin macrophages represent a target of EBV infection in dcSSc patients.

## Discussion

Monocyte/macrophage inflammation and TLR activation have been identified as possible contributors to the pathogenesis of many autoimmune diseases, including SSc. The findings presented here show that activation of TLR8 is induced by EBV/lytic genes in HD and SSc EBV-p2089-infected monocytes and that expression of TLR8 and IFN innate immune mediators is significantly increased in freshly isolated dcSSc monocytes carrying infectious EBV compared to monocytes from HDs. This is the first study that identifies infectious EBV in monocytes from patients with SSc, and mechanistically links EBV with activation of TLR8 and the IFN innate immune response in freshly isolated dcSSc monocytes. These data support a potential role for infectious EBV in contributing to chronic innate immune activation in dcSSc monocytes.

EBV has been reported to induce an innate immune response characterized by strong release of IL-8 and MCP-1 via TLR9 and TLR2 in monocytes shortly after infection [15, 23]. Likewise, specific viral programs and small non-coding RNA recognized by TLR7, TLR3, and RIG-I have been reported in infected B lymphocytes and plasmacytoid dendritic cells, suggesting that activation of the innate immune response by EBV is dependent on the viral programs carried in the infected cells [15, 34]. In support of these findings, we also observed that TLR9 and TLR3 were increased in a select few monocytes.

One explanation could be that viral replication may not occur simultaneously in all EBV-infected monocytes, and that distinct monocyte populations might also predispose different EBV viral programs in infected cells. Consistent with this observation, we found that monocytes from distinct SSc patients and HDs could be lytically or latently infected after the same exposure time to EBV. Given that the status of EBV infection is dependent on underlying changes within B lymphocytes that permit the stable maintenance of the virus genome or predispose its lytic form [35–39], it is possible that distinct monocyte populations might also influence the establishment of lytic or latent EBV infection.

The key observation that EBV DNA does not induce activation of TLR8 and/or other TLR-inflammatory responses strongly indicates that viral replication might be the prerequisite in activating the innate immune response in the infected cells.

Although immunogenicity of infectious EBV has not yet been taken into account, these findings now lead to the question of which viral-gene mRNA or viral miRNA might specifically interact with TLR8. Further investigation will be required to address this aspect.

An important aspect of this study is that almost all dcSSc and HD monocytes were able to sustain EBV replication in vitro up to 5 days PI. Expression of BZLF1, the EBV immediate early transactivator which promotes the switch from latent to lytic infection, was previously reported as early as 2 h PI with maximum expression at 20 h, suggesting that the EBV replicative cycle occurs very early in infected cells, including monocytes [21, 28, 35, 40–42]. Thus, our data extend this observation to dcSSc monocytes, showing that de novo EBV replication and activation of the IFN innate immune response could be detected up to 5 days PI.

Activation of TLR8 by synthetic ligand mimicked the EBV effects on TLR8, by inducing TLR8 and IRF7 mRNA expression. Intriguingly, selective activation of TLR8 over TLR7 has been shown to inhibit the TLR7-IFNα response, suggesting that the interaction between these two receptors may contribute to the regulation of the innate immune response [43, 44]. Consistent with the observation that TLR8 upregulation inhibits the TLR7-IFNα response, we found that expression of the BFRF1/lytic gene was associated with a trend of TLR7 downregulation in certain infected HD and dcSSc monocytes (Additional file 1: Figure S8), suggesting that activation of TLR8 may be a new strategy employed by EBV to dampen IFNα during lytic replication and control the host innate immune system.

The mechanism for EBV-induced TLR8 expression was in part dependent on the TLR8-activation pathway, since infectious EBV in the presence of bafilomycin did not completely abrogate the signaling resulting in TLR8 and IRF7 upregulation in THP-1 cells. Consistent with our findings that infectious EBV stimulates the production of TNFα mRNA (Fig. 2a) [45], which also upregulates TLR8 protein in THP-1 cells (Fig. 5b), these observations support the idea of EBV using TNFα as a mechanism to synergize with endosomal TLR8 signaling to modulate innate immune responses in infected THP-1 cells. Further investigation will be required to address this aspect.

The relevance of the IFN innate immune response and TLR8 in dcSSc patients has been substantiated by microarray analysis showing the elevated expression levels of IFN-regulated genes, chemokines, and TLR8 mRNA in ex vivo EBV-infected dcSSc monocytes compared to infected HD monocytes. The possible role of TLR8 in autoimmune diseases has been recently considered, since together with TLR7 it is one of the two TLRs expressed on the X chromosome [46]. Because most autoimmune diseases are more prevalent in females, it has been postulated that elevated levels of TLR8, as an X-linked gene, might play a direct role in the pathogenesis of these diseases, including SSc [46]. TLR8 has been implicated in the pathogenesis of arthritis, as its overexpression causes spontaneous inflammatory arthritis in mice [47]. Moreover, a recent study shows that TLR8 agonist stimulation contributes to inflammatory and profibrotic cytokine production in SSc monocytes, further supporting the link between the inflammatory/fibrosis process and TLR8 innate immune activation in the pathogenesis of SSc [48].

Different monocyte subsets may exert specific functions in response to microbes. While CD14+/CD16– classical monocytes produce more proinflammatory cytokines, including IL-6, and production of ROS, CD14–/CD16+, non-classical monocytes selectively produce high levels of TNFα in response to the same trigger [30]. We found EBV lytic proteins in ex vivo CD14+ and/or CD16+ dcSSc monocytes suggesting that EBV might infect more than one monocyte population in SSc. Therefore, it is conceivable that induction of selective cytokines may also be dependent on the monocyte population infected by EBV. In this regard, although EBV-p2089 induced several proinflammatory cytokines in HD and dcSSc monocytes in vitro, the magnitude of the induction was dissimilar among the primary cells (Fig. 2a), possibly reflecting the heterogeneity in the infected monocyte populations. It is also worth mentioning that either the innate immune response or EBV status could be affected by the phenotype of the infected monocytes. Thus, while specific monocyte subsets may not permit EBV reactivation, other populations may be more permissive to EBV replication when infected. Further studies will be needed to clarify the functional outcomes of EBV infection among distinct monocyte populations.

We show that expression of Siglec1 can be induced by EBV. Previous studies reported increased level of CD169/Sglec1 monocytes/macrophages in SSc skin, lung, and PBMCs [8, 9, 49], suggesting that CD169+ cells might contribute to the pathogenesis of SSc. While the functional role of Siglec1 in SSc is not entirely clear, it is generally accepted that CD169+ macrophages are involved in cell-cell adhesion as well as cell-pathogen interaction [50, 51]. Consistent with this observation, recent studies have shown that CD169+ macrophages were able to capture several viruses, including HIV, suggesting that sialic acid on the viral envelope facilitates HIV-I infection of macrophages through interacting with Siglec1 [52, 53]. Although the functional role of Siglec1 in EBV infection has not been taken into account, we speculate that EBV might use Siglec1 for cell-to-cell adhesion and viral spread.

Our finding suggests that Siglec1 is inclined to be more induced in SSc non-classical monocytes. This result is in agreement with previous studies which reported increased levels of CD16 and CCL2 in Siglec1-positive monocytes, suggesting that monocytes expressing Siglec1 are more activated to produce cytokines in SSc [8]. While the characterization of the EBV-infected cell population remains to be determined in vivo, it is possible that Siglec1-positive CD14–/CD16+ monocytes might carry active EBV infection. Intriguingly, non-classical monocytes have shown the ability to adhere to the endothelium in vivo and in vitro and to exert specific functions in the response to viruses via TLR7/8 [30, 32]. We speculate that EBV/Siglec1/CD14–/CD16+ monocytes might facilitate EBV dissemination in SSc tissues by permitting the adhesion of monocytes to the vascular endothelium in vivo.

Our data showed that skin macrophages expressed EBV lytic protein, further suggesting that EBV-infected monocytes might migrate into the skin and facilitate dissemination of EBV to endothelial cells and fibroblasts [13]. Although this is the first report showing the presence of EBV lytic protein in dcSSc skin macrophages, the possibility that monocytes/macrophages might serve as reservoir and/or vehicle for EBV infection in tissue sites is in agreement with a previous study [54].

## Conclusion

This study provides the first evidence of EBV/lytic gene mRNA activating the TLR8 innate immune pathway and suggests a novel mechanism mediating monocyte inflammation in SSc by which EBV triggers the innate immune response in infected cells.

## Additional files

Additional file 1: Figure S1. Experimental setup of monocyte purification and monocyte subsets in dcSSc patient and HD groups. (A): a representative cytofluorimetric analysis of CD14 and CD163 expressing monocytes pre- and post- selection from PBMCs. (B): mRNA expression of the indicated genes evaluated in the fraction of CD19+lymphocytes and CD14/CD16 monocytes from dcSSc patients and HDs by qPCR. **Figure S2.** TLRs expression in EBV lytic infected monocytes evaluated by qPCR in negative selected CD14/CD16 monocytes infected with EBV-p2089 5 days post infection (PI). **Figure S3.** Innate immune mediators are not induced in monocytes expressing the latent form of EBV infection in negative selected CD14/CD16 monocytes infected with EBV-p2089, 5 days post infection (PI). **Figure S4.** Increased expression of Siglec1 and CCL2 is equally induced by EBV lytic infection and CL075/TLR8 synthetic ligand in HD and dcSSc EBV-lytic infected monocytes. **Figure S5.** TLR8 mRNA expression in UV radiated THP-1 cells. Bars represent mean ±S.E.M. from 3 separate experiments. **Figure S6.** (A-B) Western Blot analysis of full length of TLR8 and IRF7 expression in EBV-infected/uninfected THP-1 cells. **Figure S7.** PCR products of EBV DNA in monocytes and in B-lymphocytes from representative dcSSc and HD; DNA from Raji-EBV-positive cells and DNA from 293 were used as positive control and negative control, respectively. Gapdh used as internal control. **Figure S8.** The role of BFRF1-lytic gene for selective activation of TLR8 over TLR7 gene expression in EBV-p2089 infected monocytes. mRNA expression of EBV lytic genes and TLR genes was analyzed by q-PCR. Results are expressed as fold induction normalized to each mock-infected control. 18S ribosomal RNA levels were used as internal control. (PDF 527 kb)
Additional file 2: Table S1. Real-time PCR primers used for detection of viral and host gene cDNAs. (DOCX 14 kb)
Additional file 3: Table S2. Gene expression profile of top induced genes in SSc vs HD monocytes (FDR *q* < 0.25). (DOCX 70 kb)

## Abbreviations

dcSSc: Diffuse cutaneous systemic sclerosis
EBV: Epstein-Barr virus
EBV-p2089: recombinant EBV
FDR: False discovery rate
HD: Healthy donor
IFN: Interferon
IL: Interleukin
IT: Immunosuppressive therapy
PBMC: Peripheral blood mononuclear cell
PCR: Polymerase chain reaction
PI: Post-infection
Siglec1: Sialic acid-binding Ig-like lectin 1 Siglec1/CD169
SSc: Systemic sclerosis
TLR: Toll-like receptor
TNF: Tumor necrosis factor

## References

[1] Allanore Y, Distler O (2015). Systemic sclerosis in 2014: advances in cohort enrichment shape future of trial design. Nat Rev Rheumatol.

[2] Farina GA, York MR, Di Marzio M, Collins CA, Meller S, Homey B, Rifkin IR, Marshak-Rothstein A, Radstake TR, Lafyatis R (2010). Poly(I:C) drives type I IFN- and TGFbeta-mediated inflammation and dermal fibrosis simulating altered gene expression in systemic sclerosis. J Invest Dermatol.

[3] Broen JC, Radstake TR, Rossato M (2014). The role of genetics and epigenetics in the pathogenesis of systemic sclerosis. Nat Rev Rheumatol.

[4] Huang E, Wells CA (2014). The ground state of innate immune responsiveness is determined at the interface of genetic, epigenetic, and environmental influences. J Immunol.

[5] Wermuth PJ, Jimenez SA (2015). The significance of macrophage polarization subtypes for animal models of tissue fibrosis and human fibrotic diseases. Clin Transl Med.

[6] Assassi S, Swindell WR, Wu M, Tan FD, Khanna D, Furst DE, Tashkin DP, Jahan-Tigh RR, Mayes MD, Gudjonsson JE (2015). Dissecting the heterogeneity of skin gene expression patterns in systemic sclerosis. Arthritis Rheumatol.

[7] Duan H, Fleming J, Pritchard DK, Amon LM, Xue J, Arnett HA, Chen G, Breen P, Buckner JH, Molitor JA (2008). Combined analysis of monocyte and lymphocyte messenger RNA expression with serum protein profiles in patients with scleroderma. Arthritis Rheum.

[8] York MR, Nagai T, Mangini AJ, Lemaire R, van Seventer JM, Lafyatis R (2007). A macrophage marker, Siglec-1, is increased on circulating monocytes in patients with systemic sclerosis and induced by type I interferons and toll-like receptor agonists. Arthritis Rheum.

[9] Christmann RB, Hayes E, Pendergrass S, Padilla C, Farina G, Affandi AJ, Whitfield ML, Farber HW, Lafyatis R (2011). Interferon and alternative activation of monocyte/macrophages in systemic sclerosis-associated pulmonary arterial hypertension. Arthritis Rheum.

[10] Brkic Z, van Bon L, Cossu M, van Helden-Meeuwsen CG, Vonk MC, Knaapen H, van den Berg W, Dalm VA, Van Daele PL, Severino A (2016). The interferon type I signature is present in systemic sclerosis before overt fibrosis and might contribute to its pathogenesis through high BAFF gene expression and high collagen synthesis. Ann Rheum Dis.

[11] Liu X, Mayes MD, Tan FK, Wu M, Reveille JD, Harper BE, Draeger HT, Gonzalez EB, Assassi S (2013). Correlation of interferon-inducible chemokine plasma levels with disease severity in systemic sclerosis. Arthritis Rheum.

[12] Farina A, Farina GA (2015). Fresh insights into disease etiology and the role of microbial pathogens. Curr Rheumatol Rep.

[13] Farina A, Cirone M, York M, Lenna S, Padilla C, McLaughlin S, Faggioni A, Lafyatis R, Trojanowska M, Farina GA (2014). Epstein-Barr virus infection induces aberrant TLR activation pathway and fibroblast-myofibroblast conversion in scleroderma. J Invest Dermatol.

[14] Fattal I, Shental N, Molad Y, Gabrielli A, Pokroy-Shapira E, Oren S, Livneh A, Langevitz P, Pauzner R, Sarig O (2014). Epstein-Barr virus antibodies mark systemic lupus erythematosus and scleroderma patients negative for anti-DNA. Immunology.

[15] Lunemann A, Rowe M, Nadal D (2015). Innate immune recognition of EBV. Curr Top Microbiol Immunol.

[16] LeRoy EC, Black C, Fleischmajer R, Jablonska S, Krieg T, Medsger TA, Rowell N, Wollheim F (1988). Scleroderma (systemic sclerosis): classification, subsets and pathogenesis. J Rheumatol.

[17] Farina A, Santarelli R, Gonnella R, Bei R, Muraro R, Cardinali G, Uccini S, Ragona G, Frati L, Faggioni A (2000). The BFRF1 gene of Epstein-Barr virus encodes a novel protein. J Virol.

[18] Farina A, Feederle R, Raffa S, Gonnella R, Santarelli R, Frati L, Angeloni A, Torrisi MR, Faggioni A, Delecluse HJ (2005). BFRF1 of Epstein-Barr virus is essential for efficient primary viral envelopment and egress. J Virol.

[19] Gonnella R, Farina A, Santarelli R, Raffa S, Feederle R, Bei R, Granato M, Modesti A, Frati L, Delecluse HJ (2005). Characterization and intracellular localization of the Epstein-Barr virus protein BFLF2: interactions with BFRF1 and with the nuclear lamina. J Virol.

[20] Schlitt A, Blankenberg S, Weise K, Gartner BC, Mehrer T, Peetz D, Meyer J, Darius H, Rupprecht HJ (2005). Herpesvirus DNA (Epstein-Barr virus, herpes simplex virus, cytomegalovirus) in circulating monocytes of patients with coronary artery disease. Acta Cardiol.

[21] Savard M, Gosselin J (2006). Epstein-Barr virus immunossuppression of innate immunity mediated by phagocytes. Virus Res.

[22] Lacerte P, Brunet A, Egarnes B, Duchene B, Brown JP, Gosselin J (2016). Overexpression of TLR2 and TLR9 on monocyte subsets of active rheumatoid arthritis patients contributes to enhance responsiveness to TLR agonists. Arthritis Res Ther.

[23] Fiola S, Gosselin D, Takada K, Gosselin J (2010). TLR9 contributes to the recognition of EBV by primary monocytes and plasmacytoid dendritic cells. J Immunol.

[24] McKenzie J, El-Guindy A (2015). Epstein-Barr virus lytic cycle reactivation. Curr Top Microbiol Immunol.

[25] Barnes BJ, Field AE, Pitha-Rowe PM (2003). Virus-induced heterodimer formation between IRF-5 and IRF-7 modulates assembly of the IFNA enhanceosome in vivo and transcriptional activity of IFNA genes. J Biol Chem.

[26] Forsbach A, Samulowitz U, Volp K, Hofmann HP, Noll B, Tluk S, Schmitz C, Wader T, Muller C, Podszuweit A (2011). Dual or triple activation of TLR7, TLR8, and/or TLR9 by single-stranded oligoribonucleotides. Nucleic acid therapeutics.

[27] Heil F, Hemmi H, Hochrein H, Ampenberger F, Kirschning C, Akira S, Lipford G, Wagner H, Bauer S (2004). Species-specific recognition of single-stranded RNA via toll-like receptor 7 and 8. Science.

[28] Savard M, Belanger C, Tardif M, Gourde P, Flamand L, Gosselin J (2000). Infection of primary human monocytes by Epstein-Barr virus. J Virol.

[29] Kuznik A, Bencina M, Svajger U, Jeras M, Rozman B, Jerala R (2011). Mechanism of endosomal TLR inhibition by antimalarial drugs and imidazoquinolines. J Immunol.

[30] Cros J, Cagnard N, Woollard K, Patey N, Zhang SY, Senechal B, Puel A, Biswas SK, Moshous D, Picard C (2010). Human CD14dim monocytes patrol and sense nucleic acids and viruses via TLR7 and TLR8 receptors. Immunity.

[31] Burbano C, Vasquez G, Rojas M (2014). Modulatory effects of CD14 + CD16++ monocytes on CD14++CD16– monocytes: a possible explanation of monocyte alterations in systemic lupus erythematosus. Arthritis Rheumatol.

[32] Schmidl C, Renner K, Peter K, Eder R, Lassmann T, Balwierz PJ, Itoh M, Nagao-Sato S, Kawaji H, Carninci P (2014). Transcription and enhancer profiling in human monocyte subsets. Blood.

[33] Higashi-Kuwata N, Jinnin M, Makino T, Fukushima S, Inoue Y, Muchemwa FC, Yonemura Y, Komohara Y, Takeya M, Mitsuya H (2010). Characterization of monocyte/macrophage subsets in the skin and peripheral blood derived from patients with systemic sclerosis. Arthritis Res Ther.

[34] Martin HJ, Lee JM, Walls D, Hayward SD (2007). Manipulation of the toll-like receptor 7 signaling pathway by Epstein-Barr virus. J Virol.

[35] Hislop AD, Taylor GS, Sauce D, Rickinson AB (2007). Cellular responses to viral infection in humans: lessons from Epstein-Barr virus. Annu Rev Immunol.

[36] Young LS, Arrand JR, Murray PG. EBV gene expression and regulation. In: Human Herpesviruses: Biology, Therapy, and Immunoprophylaxis. Arvin A, Campadelli-Fiume G, Mocarski E, Moore PS, Roizman B, Whitley R, Yamanishi K, editors. Cambridge: Cambridge University Press; 2007. 21348071

[37] Jones RJ, Smith LJ, Dawson CW, Haigh T, Blake NW, Young LS (2003). Epstein-Barr virus nuclear antigen 1 (EBNA1) induced cytotoxicity in epithelial cells is associated with EBNA1 degradation and processing. Virology.

[38] Taylor GS, Long HM, Brooks JM, Rickinson AB, Hislop AD (2015). The immunology of Epstein-Barr virus-induced disease. Annu Rev Immunol.

[39] Knox PG, Li QX, Rickinson AB, Young LS (1996). In vitro production of stable Epstein-Barr virus-positive epithelial cell clones which resemble the virus:cell interaction observed in nasopharyngeal carcinoma. Virology.

[40] Kalla M, Hammerschmidt W (2012). Human B cells on their route to latent infection—early but transient expression of lytic genes of Epstein-Barr virus. Eur J Cell Biol.

[41] Wu Y, Maruo S, Yajima M, Kanda T, Takada K (2007). Epstein-Barr virus (EBV)-encoded RNA 2 (EBER2) but not EBER1 plays a critical role in EBV-induced B-cell growth transformation. J Virol.

[42] Ladell K, Dorner M, Zauner L, Berger C, Zucol F, Bernasconi M, Niggli FK, Speck RF, Nadal D (2007). Immune activation suppresses initiation of lytic Epstein-Barr virus infection. Cell Microbiol.

[43] Wang J, Shao Y, Bennett TA, Shankar RA, Wightman PD, Reddy LG (2006). The functional effects of physical interactions among Toll-like receptors 7, 8, and 9. J Biol Chem.

[44] Gorden KB, Gorski KS, Gibson SJ, Kedl RM, Kieper WC, Qiu X, Tomai MA, Alkan SS, Vasilakos JP (2005). Synthetic TLR agonists reveal functional differences between human TLR7 and TLR8. J Immunol.

[45] D’Addario M, Ahmad A, Morgan A, Menezes J (2000). Binding of the Epstein-Barr virus major envelope glycoprotein gp350 results in the upregulation of the TNF-alpha gene expression in monocytic cells via NF-kappaB involving PKC, PI3-K and tyrosine kinases. J Mol Biol.

[46] Voskuhl R (2011). Sex differences in autoimmune diseases. Biol Sex Differences.

[47] Guiducci C, Gong M, Cepika AM, Xu Z, Tripodo C, Bennett L, Crain C, Quartier P, Cush JJ, Pascual V (2013). RNA recognition by human TLR8 can lead to autoimmune inflammation. J Exp Med.

[48] Ciechomska M, O’Reilly S, Przyborski S, Oakley F, Bogunia-Kubik K, van Laar JM. Histone demethylation and Toll-like Receptor 8-Dependent Cross-Talk in Monocytes Promotes Transdifferentiation of Fibroblasts in Systemic Sclerosis Via Fra-2. Arthritis Rheumatol. 2016;68(6):1493–504. doi:10.1002/art.39602. 10.1002/art.39602 26814616

[49] Farina G, Lafyatis D, Lemaire R, Lafyatis R (2010). A four-gene biomarker predicts skin disease in patients with diffuse cutaneous systemic sclerosis. Arthritis Rheum.

[50] Crocker PR, Paulson JC, Varki A (2007). Siglecs and their roles in the immune system. Nat Rev Immunol.

[51] Martinez-Pomares L, Gordon S (2012). CD169+ macrophages at the crossroads of antigen presentation. Trends Immunol.

[52] Chavez-Galan L, Olleros ML, Vesin D, Garcia I (2015). Much more than M1 and M2 macrophages, there are also CD169(+) and TCR(+) macrophages. Front Immunol.

[53] Sewald X, Ladinsky MS, Uchil PD, Beloor J, Pi R, Herrmann C, Motamedi N, Murooka TT, Brehm MA, Greiner DL (2015). Retroviruses use CD169-mediated trans-infection of permissive lymphocytes to establish infection. Science.

[54] Tugizov S, Herrera R, Veluppillai P, Greenspan J, Greenspan D, Palefsky JM (2007). Epstein-Barr virus (EBV)-infected monocytes facilitate dissemination of EBV within the oral mucosal epithelium. J Virol.

