# Supplementary figures and images for: Epstein-Barr virus lytic infection promotes activation of Toll-like receptor 8 innate immune response in systemic sclerosis monocytes
Source: Arthritis Res Ther. 2017 Feb 28;19:39. doi: 10.1186/s13075-017-1237-9 (PMC5331713; doi:10.1186/s13075-017-1237-9)

Figure S1

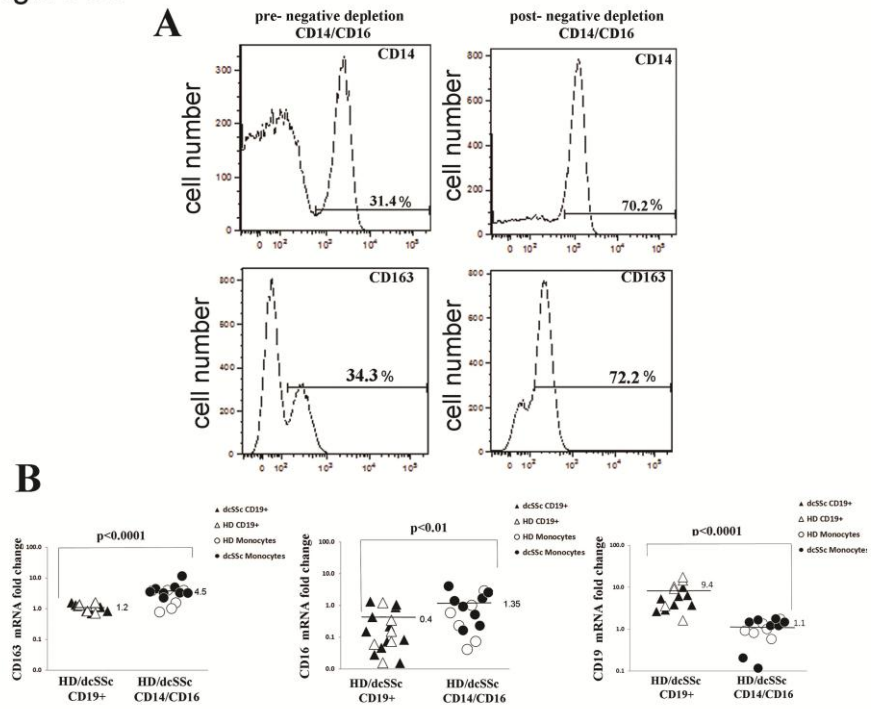

Figure S2

A

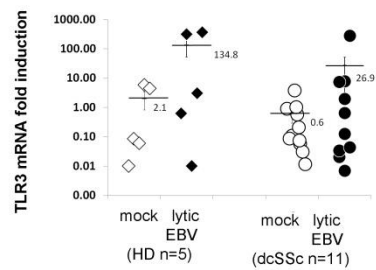

B

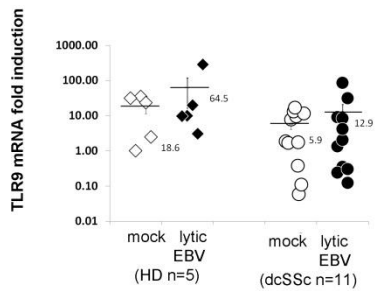

C

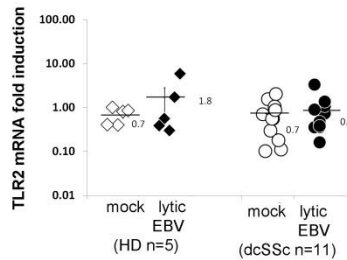

**Figure S3**

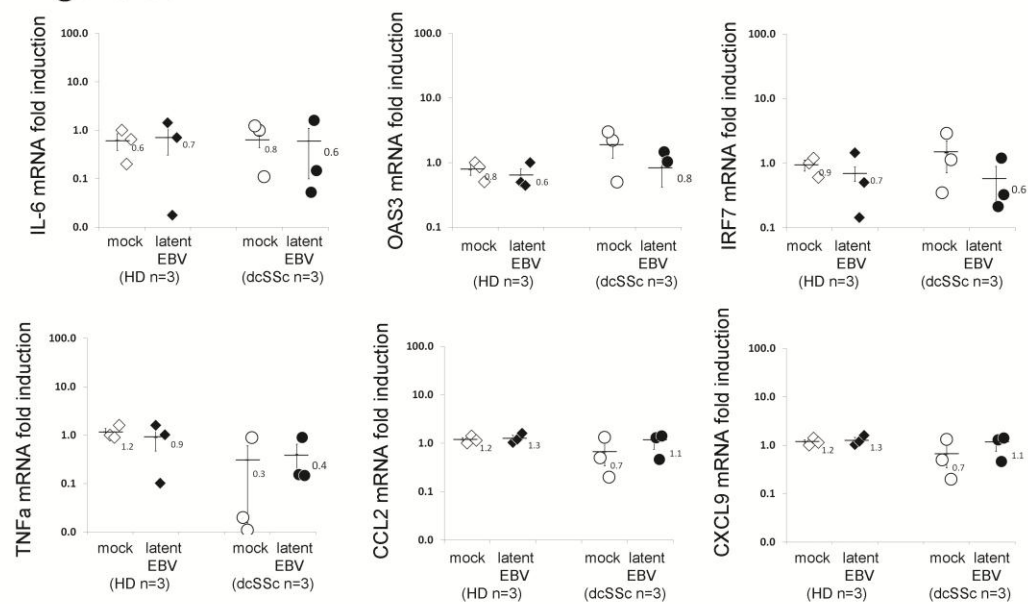

Figure S4

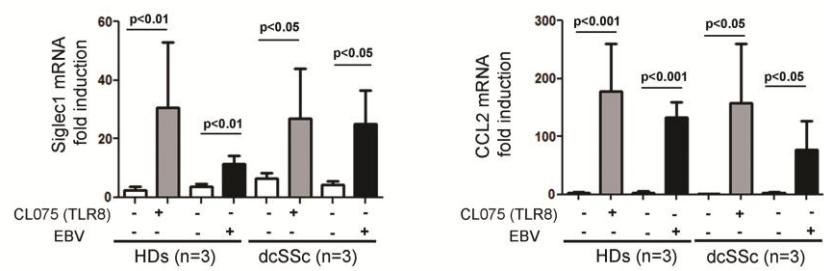

Figure S5

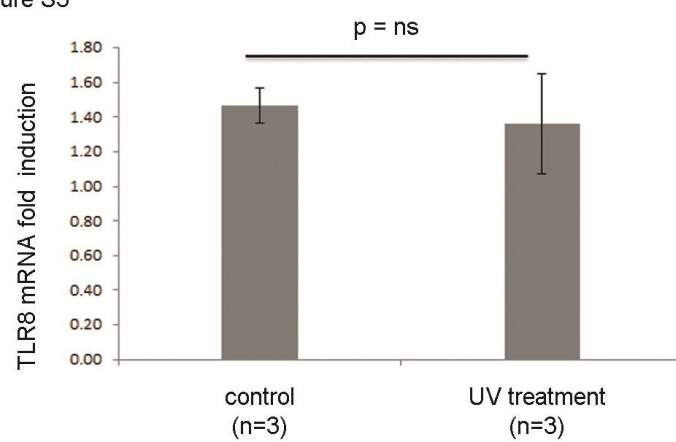

Figure S6

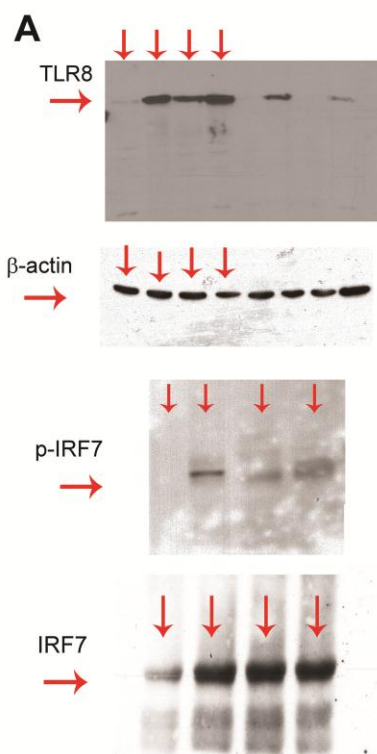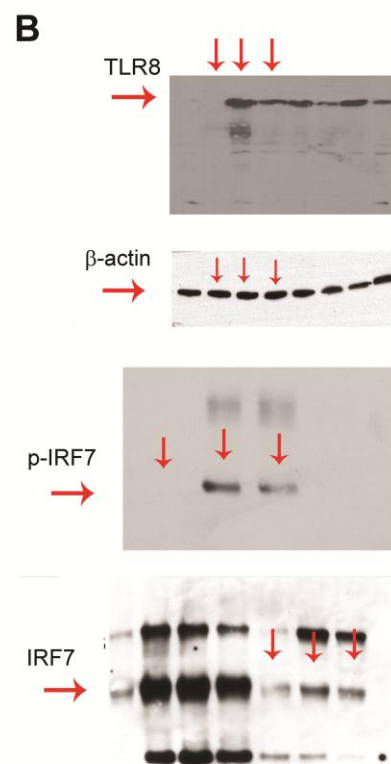

Figure S7

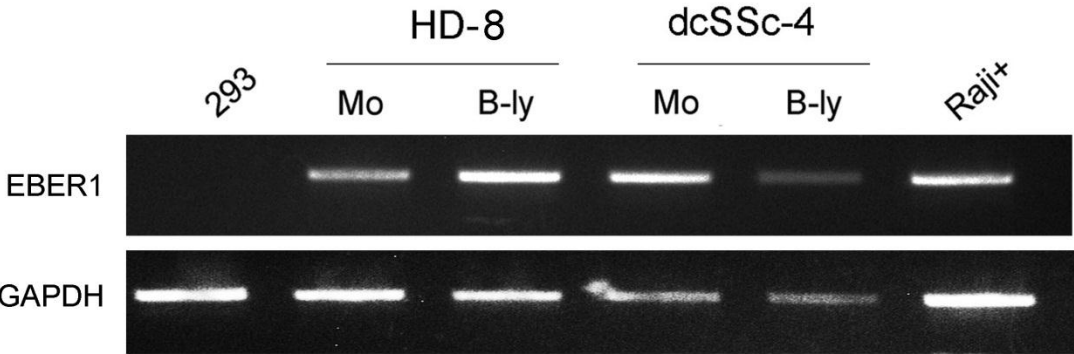

Figure S8

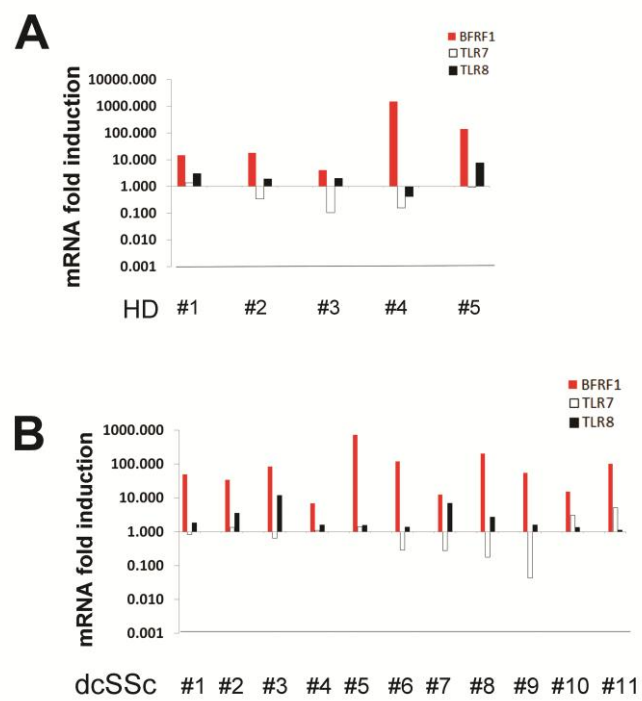

Supplement: Additional file 1: Figure S1. — Experimental setup of monocyte purification and monocyte subsets in dcSSc patient and HD groups. (A): a representative cytofluorimetric analysis of CD14 and CD163 expressing monocytes pre- and post- selection from PBMCs. (B): mRNA expression of the indicated genes evaluated in the fraction of CD19+lymphocytes and CD14/CD16 monocytes from dcSSc patients and HDs by qPCR. Figure S2. TLRs expression in EBV lytic infected monocytes evaluated by qPCR in negative selected CD14/CD16 monocytes infected with EBV-p2089 5 days post infection (PI). Figure S3. Innate immune mediators are not induced in monocytes expressing the latent form of EBV infection in negative selected CD14/CD16 monocytes infected with EBV-p2089, 5 days post infection (PI). Figure S4. Increased expression of Siglec1 and CCL2 is equally induced by EBV lytic infection and CL075/TLR8 synthetic ligand in HD and dcSSc EBV-lytic infected monocytes. Figure S5. TLR8 mRNA expression in UV radiated THP-1 cells. Bars represent mean ±S.E.M. from 3 separate experiments. Figure S6. (A-B) Western Blot analysis of full length of TLR8 and IRF7 expression in EBV-infected/uninfected THP-1 cells. Figure S7. PCR products of EBV DNA in monocytes and in B-lymphocytes from representative dcSSc and HD; DNA from Raji-EBV-positive cells and DNA from 293 were used as positive control and negative control, respectively. Gapdh used as internal control. Figure S8. The role of BFRF1-lytic gene for selective activation of TLR8 over TLR7 gene expression in EBV-p2089 infected monocytes. mRNA expression of EBV lytic genes and TLR genes was analyzed by q-PCR. Results are expressed as fold induction normalized to each mock-infected control. 18S ribosomal RNA levels were used as internal control. (PDF 527 kb) [file 13075_2017_1237_MOESM1_ESM.pdf]
