# Supplementary material for: Epstein-Barr virus lytic infection promotes activation of Toll-like receptor 8 innate immune response in systemic sclerosis monocytes
Source: Arthritis Res Ther. 2017 Feb 28;19:39. doi: 10.1186/s13075-017-1237-9 (PMC5331713; doi:10.1186/s13075-017-1237-9)
Supplement: Additional file 2: Table S1. — Real-time PCR primers used for detection of viral and host gene cDNAs. (DOCX 14 kb) [file 13075_2017_1237_MOESM2_ESM.docx]

Table S1. Real-time PCR primers used for detection of viral and host gene cDNAs.

| **GENE** | Forward (5’-3’) | Reverse (5’-3’) |
| --- | --- | --- |
| **qPCR** |  |  |
| 18S | -CGGCTACCACATCCAAGGAA- | -AGCTGGAATTACCGCGGC- |
| BFRF1 | -AACACCGGACGACGATGC - | -GCGAGGCCTTCTGGGC- |
| BLLF1 | -CATTGGTAGCCGTTCGTGTGATAAT- | -GCGAGCAATCGGACATTTGACAT- |
| TLR8 | -TTATGTGTTCCAGGAACTCAGAGAA- | -TAATACCCAAGTTGTCGATAAGTTTG- |
| CD163 | -CAAGATGCTGGCGTGACAT- | -GCTGCCTCCACCTCTAAGTC- |
| FCGR3A (CD16a) | -AGGGGGCTTTTTGGGAGTA- | -TGATGAGATGGTTGACACTGC- |
