# Supplementary material for: Epstein-Barr virus lytic infection promotes activation of Toll-like receptor 8 innate immune response in systemic sclerosis monocytes
Source: Arthritis Res Ther. 2017 Feb 28;19:39. doi: 10.1186/s13075-017-1237-9 (PMC5331713; doi:10.1186/s13075-017-1237-9)
Supplement: Additional file 3: Table S2. — Gene expression profile of top induced genes in SSc vs HD monocytes (FDR q < 0.25). (DOCX 70 kb) [file 13075_2017_1237_MOESM3_ESM.docx]

**Table S2**: Gene expression profile of top induced genes in SSc vs HD monocytes.

| **Gene symbol** | **Gene name** | **fold change** |
| --- | --- | --- |
| DEFA3 | defensin, alpha 3, neutrophil-specific | 20.4 |
| IFI44 | interferon-induced protein 44 | 2.1 |
| C6orf211 | chromosome 6 open reading frame 211 | 2.1 |
| MAGED2 | melanoma antigen family D, 2 | 1.6 |
| IFI44L | interferon-induced protein 44-like | 2.7 |
| RDBP | RD RNA binding protein | 2.0 |
| MRPS18C | mitochondrial ribosomal protein S18C | 2.2 |
| RSAD2 | radical S-adenosyl methionine domain containing 2 | 2.6 |
| BST2 | bone marrow stromal cell antigen 2 | 2.8 |
| NFE2L3 | nuclear factor (erythroid-derived 2)-like 3 | 2.1 |
| MRPL24 | mitochondrial ribosomal protein L24 | 1.9 |
| SLPI | secretory leukocyte peptidase inhibitor | 1.9 |
| APOO | apolipoprotein O | 2.0 |
| SDC4 | syndecan 4 | 2.5 |
| TNFSF12 | tumor necrosis factor (ligand) superfamily, member 12 | 1.7 |
| ISG15 | ISG15 ubiquitin-like modifier | 1.9 |
| CCDC115 | coiled-coil domain containing 115 | 2.0 |
| ORMDL2 | ORM1-like 2 (S. cerevisiae) | 1.8 |
| PPP1R7 | protein phosphatase 1, regulatory subunit 7 | 1.6 |
| SPATS2L | spermatogenesis associated, serine-rich 2-like | 1.7 |
| CMPK2 | cytidine monophosphate (UMP-CMP) kinase 2, mitochondrial | 2.1 |
| IFIT1 | interferon-induced protein with tetratricopeptide repeats 1 | 2.5 |
| IFI6 | interferon, alpha-inducible protein 6 | 2.5 |
| CTSC | cathepsin C | 1.8 |
| OAS2 | 2'-5'-oligoadenylate synthetase 2, 69/71kDa | 1.9 |
| ARPC4 | actin related protein 2/3 complex, subunit 4, 20kDa | 1.5 |
| CCR1 | chemokine (C-C motif) receptor 1 | 2.6 |
| IFIT2 | interferon-induced protein with tetratricopeptide repeats 2 | 2.1 |
| EPSTI1 | epithelial stromal interaction 1 (breast) | 2.1 |
| C20orf43 | chromosome 20 open reading frame 43 | 1.4 |
| HBXIP | hepatitis B virus x interacting protein | 1.7 |
| SDF2L1 | stromal cell-derived factor 2-like 1 | 1.7 |
| MS4A7 | membrane-spanning 4-domains, subfamily A, member 7 | 1.8 |
| HTATIP2 | HIV-1 Tat interactive protein 2, 30kDa | 1.5 |
| LOC100507463 | uncharacterized LOC100507463 | 1.7 |
| CNP | 2',3'-cyclic nucleotide 3' phosphodiesterase | 1.5 |
| MRPL13 | mitochondrial ribosomal protein L13 | 1.6 |
| ELANE | elastase, neutrophil expressed | 3.0 |
| LAP3 | leucine aminopeptidase 3 | 1.7 |
| IFIT3 | interferon-induced protein with tetratricopeptide repeats 3 | 2.6 |
| DNLZ | DNL-type zinc finger | 1.8 |
| STK32B | serine/threonine kinase 32B | 1.6 |
| SCARNA9 | small Cajal body-specific RNA 9 | 2.7 |
| CEACAM8 | carcinoembryonic antigen-related cell adhesion molecule 8 | 5.0 |
| PLEKHO1 | pleckstrin homology domain containing, family O member 1 | 1.6 |
| AHSA1 | AHA1, activator of heat shock 90kDa protein ATPase homolog 1 (yeast) | 1.6 |
| C2 | complement component 2 | 2.0 |
| PTTG1IP | pituitary tumor-transforming 1 interacting protein | 1.7 |
| IFIT5 | interferon-induced protein with tetratricopeptide repeats 5 | 1.6 |
| EID2 | EP300 interacting inhibitor of differentiation 2 | 1.4 |
| PRTN3 | proteinase 3 | 2.1 |
| RPS27L | ribosomal protein S27-like | 1.5 |
| ELOF1 | elongation factor 1 homolog (S. cerevisiae) | 2.2 |
| LOC729013 | uncharacterized LOC729013 | 1.8 |
| LMO2 | LIM domain only 2 (rhombotin-like 1) | 1.8 |
| AATF | apoptosis antagonizing transcription factor | 1.5 |
| IK | IK cytokine, down-regulator of HLA II | 1.6 |
| PMVK | phosphomevalonate kinase | 1.8 |
| ABCB8 | ATP-binding cassette, sub-family B (MDR/TAP), member 8 | 1.4 |
| BTN3A2 | butyrophilin, subfamily 3, member A2 | 1.4 |
| SERPING1 | serpin peptidase inhibitor, clade G (C1 inhibitor), member 1 | 3.6 |
| BCAP31 | B-cell receptor-associated protein 31 | 1.5 |
| SNX12 | sorting nexin 12 | 1.5 |
| LAMTOR2 | late endosomal/lysosomal adaptor, MAPK and MTOR activator 2 | 1.5 |
| PCTP | phosphatidylcholine transfer protein | 1.8 |
| P2RX4 | purinergic receptor P2X, ligand-gated ion channel, 4 | 1.5 |
| OAZ2 | ornithine decarboxylase antizyme 2 | 2.5 |
| PSMC5 | proteasome (prosome, macropain) 26S subunit, ATPase, 5 | 1.8 |
| ODF3B | outer dense fiber of sperm tails 3B | 1.6 |
| CHMP5 | charged multivesicular body protein 5 | 1.5 |
| GPKOW | G patch domain and KOW motifs | 1.5 |
| MS4A3 | membrane-spanning 4-domains, subfamily A, member 3 (hematopoietic cell-specific) | 4.2 |
| MRPL50 | mitochondrial ribosomal protein L50 | 1.5 |
| TRAPPC6A | trafficking protein particle complex 6A | 1.8 |
| AZU1 | azurocidin 1 | 1.9 |
| DCLRE1B | DNA cross-link repair 1B | 2.0 |
| **CXCL9*** | chemokine (C-X-C motif) ligand 9 | 6.1 |
| MVP | major vault protein | 1.5 |
| THYN1 | thymocyte nuclear protein 1 | 1.5 |
| FAM32A | family with sequence similarity 32, member A | 1.7 |
| ABHD4 | abhydrolase domain containing 4 | 1.6 |
| WDR18 | WD repeat domain 18 | 1.5 |
| **LY6E*** | lymphocyte antigen 6 complex, locus E | 1.8 |
| HERC5 | HECT and RLD domain containing E3 ubiquitin protein ligase 5 | 1.9 |
| MGAT1 | mannosyl (alpha-1,3-)-glycoprotein beta-1,2-N-acetylglucosaminyltransferase | 1.7 |
| **OAS3*** | 2'-5'-oligoadenylate synthetase 3, 100kDa | 2.0 |
| LY96 | lymphocyte antigen 96 | 1.9 |
| TMX2 | thioredoxin-related transmembrane protein 2 | 1.7 |
| DDRGK1 | DDRGK domain containing 1 | 1.5 |
| YBEY | ybeY metallopeptidase (putative) | 1.6 |
| LYRM1 | LYR motif containing 1 | 1.4 |
| PFDN6 | prefoldin subunit 6 | 1.6 |
| ANXA3 | annexin A3 | 3.0 |
| RNF25 | ring finger protein 25 | 1.5 |
| ARF3 | ADP-ribosylation factor 3 | 1.5 |
| TICAM1 | toll-like receptor adaptor molecule 1 | 1.5 |
| RRAGA | Ras-related GTP binding A | 1.4 |
| BANF1 | barrier to autointegration factor 1 | 1.6 |
| APOL1 | apolipoprotein L, 1 | 1.9 |
| IFITM3 | interferon induced transmembrane protein 3 | 1.7 |
| VTI1B | vesicle transport through interaction with t-SNAREs homolog 1B (yeast) | 1.5 |
| GBA | glucosidase, beta, acid | 1.7 |
| STK16 | serine/threonine kinase 16 | 1.5 |
| ELP5 | elongator acetyltransferase complex subunit 5 | 1.6 |
| KRT14 | keratin 14 | 1.8 |
| HMGN5 | high mobility group nucleosome binding domain 5 | 1.7 |
| IFI35 | interferon-induced protein 35 | 2.0 |
| GPR33 | G protein-coupled receptor 33 (gene/pseudogene) | 1.4 |
| ARRDC4 | arrestin domain containing 4 | 1.8 |
| BAIAP2-AS1 | BAIAP2 antisense RNA 1 | 1.6 |
| ING4 | inhibitor of growth family, member 4 | 1.4 |
| ATP6V0E1 | ATPase, H+ transporting, lysosomal 9kDa, V0 subunit e1 | 1.5 |
| C21orf59 | chromosome 21 open reading frame 59 | 1.4 |
| MPND | MPN domain containing | 1.4 |
| OASL | 2'-5'-oligoadenylate synthetase-like | 1.9 |
| MGST3 | microsomal glutathione S-transferase 3 | 1.5 |
| PDE7B | phosphodiesterase 7B | 1.6 |
| CDIPT | CDP-diacylglycerol--inositol 3-phosphatidyltransferase | 1.6 |
| NUCB1 | nucleobindin 1 | 1.8 |
| ANP32E | acidic (leucine-rich) nuclear phosphoprotein 32 family, member E | 1.6 |
| PPP2R4 | protein phosphatase 2A activator, regulatory subunit 4 | 1.6 |
| NDUFAF4 | NADH dehydrogenase (ubiquinone) complex I, assembly factor 4 | 1.4 |
| LOC100996249 | uncharacterized LOC100996249 | 1.7 |
| CTSL1 | cathepsin L1 | 5.4 |
| DEFA4 | defensin, alpha 4, corticostatin | 4.6 |
| TMEM39B | transmembrane protein 39B | 1.5 |
| APOL6 | apolipoprotein L, 6 | 1.4 |
| C1orf212 | chromosome 1 open reading frame 212 | 1.3 |
| TSR2 | TSR2, 20S rRNA accumulation, homolog (S. cerevisiae) | 1.9 |
| DPAGT1 | dolichyl-phosphate (UDP-N-acetylglucosamine) N-acetylglucosaminephosphotransferase 1 (GlcNAc-1-P transferase) | 1.6 |
| MRPS14 | mitochondrial ribosomal protein S14 | 1.4 |
| C1QA | complement component 1, q subcomponent, A chain | 1.9 |
| BAX | BCL2-associated X protein | 1.4 |
| LOC401397 | uncharacterized LOC401397 | 1.8 |
| RFK | riboflavin kinase | 1.7 |
| YIPF3 | Yip1 domain family, member 3 | 1.7 |
| PTGER2 | prostaglandin E receptor 2 (subtype EP2), 53kDa | 1.5 |
| TSSC4 | tumor suppressing subtransferable candidate 4 | 1.6 |
| C3 | complement component 3 | 2.2 |
| DNAJC8 | DnaJ (Hsp40) homolog, subfamily C, member 8 | 1.5 |
| SNORD34 | small nucleolar RNA, C/D box 34 | 1.6 |
| LOC100294145 | uncharacterized LOC100294145 | 1.5 |
| C2orf49 | chromosome 2 open reading frame 49 | 1.6 |
| SLC22A18 | solute carrier family 22, member 18 | 1.5 |
| FKBP2 | FK506 binding protein 2, 13kDa | 2.3 |
| MRPL15 | mitochondrial ribosomal protein L15 | 1.7 |
| DEM1 | defects in morphology 1 homolog (S. cerevisiae) | 1.7 |
| RAB39A | RAB39A, member RAS oncogene family | 1.9 |
| MED11 | mediator complex subunit 11 | 1.7 |
| CLN5 | ceroid-lipofuscinosis, neuronal 5 | 1.4 |
| USF1 | upstream transcription factor 1 | 1.6 |
| SPR | sepiapterin reductase (7,8-dihydrobiopterin:NADP+ oxidoreductase) | 1.6 |
| TMEM219 | transmembrane protein 219 | 1.5 |
| UBE2MP1 | ubiquitin-conjugating enzyme E2M pseudogene 1 | 1.7 |
| AAMP | angio-associated, migratory cell protein | 1.5 |
| PEX19 | peroxisomal biogenesis factor 19 | 1.4 |

***:** genes validated by qPCR
